# Risk factors for hospitalized patients with resistant or multidrug-resistant *Pseudomonas aeruginosa* infections: a systematic review and meta-analysis

**DOI:** 10.1186/s13756-018-0370-9

**Published:** 2018-07-04

**Authors:** Gowri Raman, Esther E. Avendano, Jeffrey Chan, Sanjay Merchant, Laura Puzniak

**Affiliations:** 1 Center for Clinical Evidence Synthesis, Tufts Medical Center, 800 Washington Street, Box 63, Boston, MA 02111 USA; 2 Merck & Co., Inc., Kenilworth, NJ USA

**Keywords:** Resistant, Multi-drug resistant, Pseudomonas aeruginosa, Risk factors, Acquisition

## Abstract

**Background:**

Identifying risk factors predicting acquisition of resistant *Pseudomonas aeruginosa* will aid surveillance and diagnostic initiatives and can be crucial in early and appropriate antibiotic therapy. We conducted a systematic review examining risk factors of acquisition of resistant *P. aeruginosa *among hospitalized patients.

**Methods:**

MEDLINE®, EMBASE®, and Cochrane Central were searched between 2000 and 2016 for studies examining independent risk factors associated with acquisition of resistant *P. aeruginosa*, among hospitalized patients. Random effects model meta-analysis was conducted when at least three or more studies were sufficiently similar.

**Results:**

Of the 54 eligible articles, 28 publications (31studies) examined multi-drug resistant (MDR) or extensively drug resistant (XDR) *P. aeruginosa* and 26 publications (29 studies) examined resistant *P. aeruginosa.* The acquisition of MDR *P. aeruginosa*, as compared with non-MDR *P. aeruginosa*, was significantly associated with intensive care unit (ICU) admission (3 studies: summary adjusted odds ratio [OR] 2.2) or use of quinolones (4 studies: summary adjusted OR 3.59). Acquisition of MDR or XDR compared with susceptible *P. aeruginosa* was significantly associated with prior hospital stay (4 studies: summary adjusted OR 1.90), use of quinolones (3 studies: summary adjusted OR 4.34), or use of carbapenems (3 studies: summary adjusted OR 13.68). The acquisition of MDR *P. aeruginosa* compared with non-*P. aeruginosa* was significantly associated with prior use of cephalosporins (3 studies: summary adjusted OR 3.96), quinolones (4 studies: summary adjusted OR 2.96), carbapenems (6 studies: summary adjusted OR 2.61), and prior hospital stay (4 studies: summary adjusted OR 1.74). The acquisition of carbapenem-resistant *P. aeruginosa* compared with susceptible *P. aeruginosa*, was statistically significantly associated with prior use of piperacillin-tazobactam (3 studies: summary adjusted OR 2.64), vancomycin (3 studies: summary adjusted OR 1.76), and carbapenems (7 studies: summary adjusted OR 4.36).

**Conclusions:**

Prior use of antibiotics and prior hospital or ICU stay was the most significant risk factors for acquisition of resistant *P. aeruginosa*. These findings provide guidance in identifying patients that may be at an elevated risk for a resistant infection and emphasize the importance of antimicrobial stewardship and infection control in hospitals.

**Electronic supplementary material:**

The online version of this article (10.1186/s13756-018-0370-9) contains supplementary material, which is available to authorized users.

## Background

There is an alarming increase in antibiotic-resistant Gram-negative infections [1–3]. Among Gram-negative infections, *Pseudomonas aeruginosa*is is one of the most common gram-negative bacteria causing nosocomial and healthcare-associated infections (HAIs) in hospitalized patients [4]. The World Health Organization places carbapenem-resistant *P. aeruginosa* as a critical priority pathogen that desperately requires new treatment options [5]. Increasing rates of multidrug-resistant (MDR) *P. aeruginosa* in HAIs and among hospitalized patients is a major public health problem [6]. MDR *P. aeruginosa* infections in the hospital setting are associated with poor outcomes including increased resource utilization and costs, morbidity, and mortality [7].

MDR *P. aeruginosa* account for 13–19% of HAIs each year in the US. The increasing level of resistance in MDR *P. aeruginosa* is often attributed to patient-to-patient transmission of resistant strains as well as newly acquired resistance owing to previous antibiotic exposure. As per standardized international terminology, MDR is defined as non-susceptibility to at least one agent in three or more antimicrobial categories and XDR is defined as non-susceptibility to at least one agent in all but two or fewer antimicrobial categories (i.e. bacterial isolates remain susceptible to only one or two categories) [8]. In severe systemic infections, consideration of MDR/XDR *P. aeruginosa* when selecting treatment is warranted to ensure timely and appropriate initial therapy. Lack of effective antibacterial therapies against *P. aeruginosa* infections severely limit effective therapeutic options and consequently, lead to inappropriate initial therapy that adversely impacts health outcomes [9]. Typically it takes 48 h to definitively identify MDR *P. aeruginosa,* which can be a detrimental delay for these patients.

There are considerable gaps and inconsistencies in knowledge regarding risk factors associated with the occurrence of MDR *P. aeruginosa* in nosocomial infections and HAIs. Identifying risk factors predicting acquisition of MDR *P. aeruginosa* or identifying subgroups of patients who are at an increased risk for acquisition of MDR *P. aeruginosa* in the hospital setting will assist in providing timely and appropriate treatment. While a recent review examined the risk factors independently associated with extensively drug-resistant (XDR) *P. aeruginosa*, there has not been a comprehensive analysis of contemporary literature reporting all levels of resistance (MDR or XDR or resistant) of *P. aeruginosa* infections [6]. This systematic review of recent published literature evaluates risk factors that are independently associated with acquisition of microbiologically identified MDR or XDR, or resistant (single drug class) *P. aeruginosa* from various sites among inpatients, hospitalized in any of the following setting including wards, intensive care units, and other types of inpatient settings.

## Methods

We performed a systematic search in the MEDLINE, Cochrane Library, and EMBASE databases for citations indexed from January 01, 2000 through December 31, 2016. The initial search strategy (Additional file 1: Table S1) includes terms related to the pathogen (*P. aeruginosa*), mode of infection (nosocomial, hospital-acquired, healthcare-acquired, hospital-associated, healthcare-associated), and risk factor assessment (risk factors, predict, risk score, risk assessment, and multivariate analysis). Additionally, search terms for Gram-negative infections, beta-lactamases, and metallo-beta-lactamases attributed to carbapenem resistance were included to increase the yield of studies with resistant (single drug class) *P. aeruginosa*. These three databases were searched because they index most of the published citations. To supplement this search, we reviewed reference lists of eligible studies using an iterative process to maximize inclusion of relevant data. We did not perform grey literature searches or systematically search for unpublished data. We reviewed abstracts from conference proceedings if they were indexed in the three aforementioned databases.

### Study definitions

Our primary objective was to examine studies evaluating risk factors associated with acquisition of MDR or XDR, or resistant *P. aeruginosa* among inpatient adult patients evaluated in the hospital setting. In this review, the hospital setting comprises all types of units, including intensive care units, emergency room/casualty, or other wards where patients were located at the time of collection of the resistant *P. aeruginosa* microbiological specimen and underwent further treatment in the hospital setting. The acquisition of resistant *P. aeruginosa* could have been either community- or hospital-acquired, but all patients must have been evaluated and treated in the hospital setting. We accepted MDR and XDR *P. aeruginosa* as defined by individual studies, although we noted considerable heterogeneity in the definitions. Non-MDR *P. aeruginosa* was also defined by the individual studies and varied in definition to include *P. aeruginosa* infections other than MDR such as susceptible *P. aeruginosa* (susceptible to all antipseudomonal agents) or resistant to any one class of drug. Non- *P. aeruginosa* was also defined by the individual studies and varied in definition to include pathogens other than *P. aeruginosa.*

### Eligibility criteria

Studies evaluating patients hospitalized in a ward, intensive or critical care unit, or any other inpatient healthcare setting (including chronic care facilities), were eligible for inclusion. Studies examining patients with either hospital-acquired or community-acquired were included, provided that they were evaluated in a hospital /inpatient setting. Risk factors that predict acquisition of MDR or XDR, or resistant *P. aeruginosa* were included. Risk factors were categorized into patient characteristics, hospital characteristics, and treatment characteristics.

The exposure and comparators of interest included: MDR or XDR *P. aeruginosa* versus resistant *P. aeruginosa* (to any one class of antibiotic); MDR or XDR *P. aeruginosa* versus susceptible *P. aeruginosa;* MDR or XDR *P. aeruginosa* versus control (any other Gram-negative pathogen or any infection with unspecified pathogen); resistant *P. aeruginosa* versus susceptible *P. aeruginosa.* The outcome of interest was microbiologically confirmed acquisition of MDR or XDR, or resistant *P. aeruginosa.* For studies that reported both the outcome of acquisition of MDR or XDR *P. aeruginosa* and single-drug resistant *P. aeruginosa,* we included the study only once (for the outcome of acquisition of MDR or XDR *P. aeruginosa*) in the analysis. We accepted all study designs except non-comparative studies, case reports, and case series. Studies that reported multivariable results adjusting for any potential confounders were eligible. No minimum study duration or follow-up time was required for inclusion.

We excluded studies of mixed infections that had more than 20% Gram-positive infections (to ensure most (80%) of the evaluated infections in eligible studies were Gram-negative infections) and studies solely examining *P. aeruginosa* resistant due to metallo-beta-lactamases. Studies published in languages other than English were excluded.

All citations identified by literature searches were independently screened by two researchers. Upon the start of citation screening, we implemented a training session where all researchers screened the same articles and conflicts were discussed. We iteratively continued training until we reached consensus regarding the nuances of the eligibility criteria for screening. During double-screening, we resolved conflicts through group discussions.

We assessed the methodological quality of each study based on predefined criteria using the Agency for Healthcare Research and Quality (AHRQ) and the New-Castle Ottawa risk of bias tool, which probes risk of selection, performance, detection, attrition, reporting, and other potential biases. Each study was extracted by one experienced methodologist (GR, EA, and JC). The extraction was reviewed and confirmed by at least one other methodologist. Any disagreements were resolved by discussion amongst the study team. Data were extracted into customized Microsoft Excel™ forms. We tested the data extraction forms on several studies and revised as necessary before full data extraction. Extracted data included variables addressing population characteristics, including severity of illness, description of exposure and comparator groups, sites of infection, outcome definitions, enrolled and analyzed sample sizes, study design features, and multivariate results. Any missing or unavailable data were deemed as not reported information. We performed random effects model meta-analyses of eligible studies if at least three or more studies were sufficiently similar in population, exposure, and outcomes [10]. If appropriate, we also conducted meta-regression analyses to evaluate study features explaining potential heterogeneity. However, due to the presence of substantial clinical heterogeneity, we also qualitatively compared results across studies (for example, source of infection).

All analyses were performed in Stata version 14 (StataCorp, College Station, Texas) with the metan, metareg, and metabias functions. We tested between study heterogeneity with the Q statistic (significant when *p* < 0.10) and quantified the extent of heterogeneity with the I^2^ statistic. It is common practice to interpret I^2^ > 50% as representing substantial inconsistency or significant statistical heterogeneity [11].

When applicable, results were also presented in table format. All meta-analyses of adjusted or multivariate results were presented in forest plots. The summary results examining the relationship between risk factors and acquisition of MDR or XDR, or resistant *P. aeruginosa* were presented as adjusted odds ratio or relative risks with 95% confidence intervals.

## Results

**Fig. 1:**
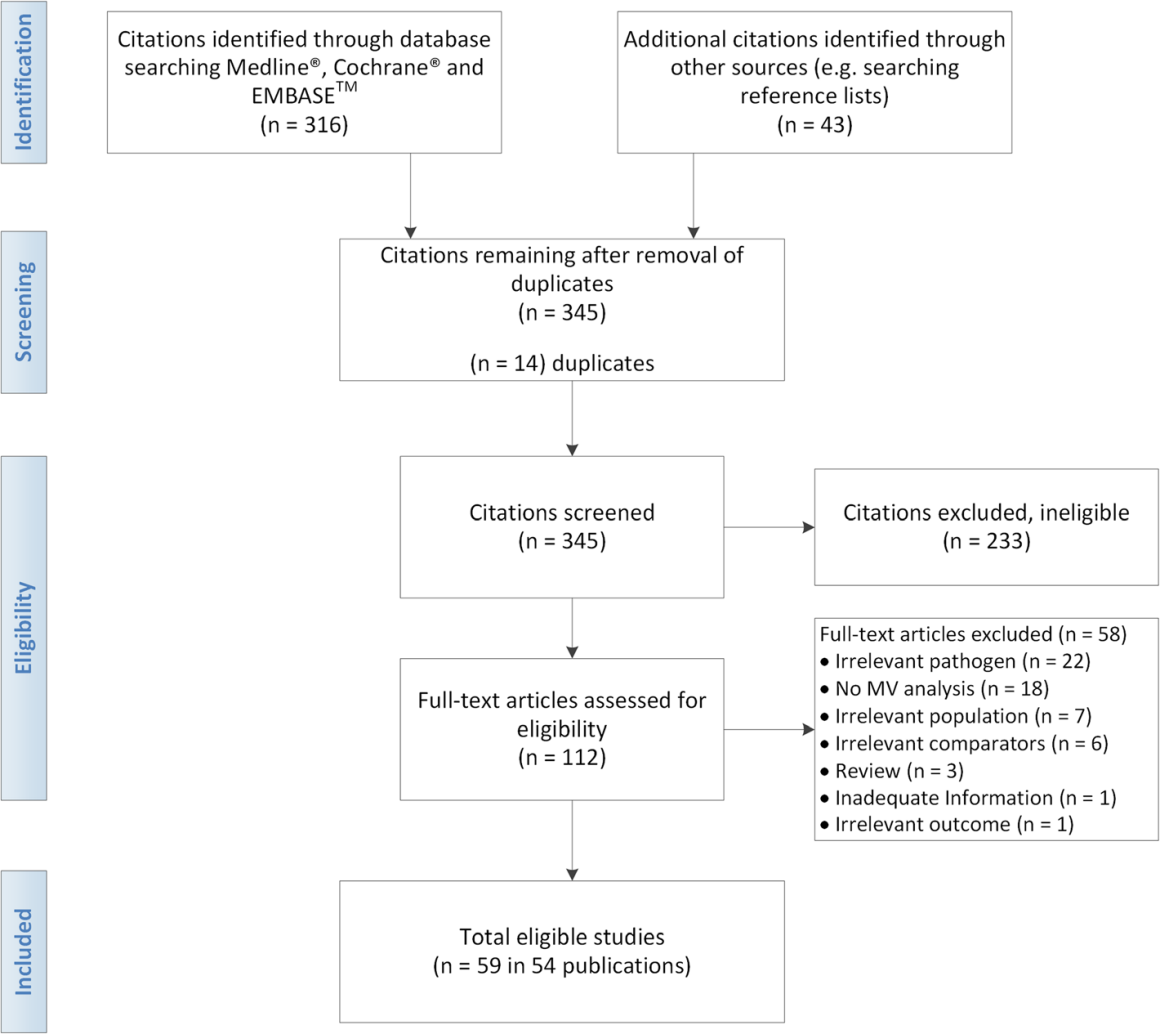
Study flow diagram

The literature search identified 316 citations and an additional 43 citations were identified through bibliographic searches. After removing duplicates, 345 abstracts were eligible for screening. Using a low threshold of eligibility criteria for review of abstracts, 112 full-text articles were retrieved. After full-text review, an additional 58 articles were excluded based on the eligibility criteria (Fig. 1). Of the 54 eligible articles, 28 articles (31 studies) examined MDR or XDR *P. aeruginosa* [12–39] and the remaining 26 articles (29 studies) examined resistant *P. aeruginosa* [41–65].

### Studies of MDR and XDR *P. aeruginosa*

Of the 28 articles (*n* = 8935 subjects) that examined risk factors for acquisition of MDR or XDR *P. aeruginosa* [12–39], nine were prospective studies and 19 were retrospective or unmatched case-control or matched case-control studies. In 31 studies from 28 articles; two articles reported two different control groups [20, 27] and one study reported two different cases [33]. The studies were conducted in Western European countries (16 studies), the USA (3 studies), the USA and Western Europe (2 studies), Asia (6 studies), Turkey (1 study), or Brazil (1 study); 3 studies were conducted in an unclear location (Table 1). Of the 28 articles, three were multi-center studies [26, 32, 35]. Twenty-one were conducted in tertiary care or university hospitals and the other 9 were conducted in any hospital.

**Table 1:** Baseline characteristics of studies in MDR or XDR*P.aeruginosa*

Author, Year	Design	Country	Case/Exposure	Control/Comparator	Total N	Age cases (Yr)	Age control (Yr)	Male cases (%)	Male control (%)
Aloush, 2006 [12]	CC, matched	Israel	MDR *P. aeruginosa*	Patients with MDR *P. aeruginosa*	164	65	63	60	50
Bodro, 2015 [13]	P, cohort	Spain	XDR *P. aeruginosa*	Other pathogens	318	Median: 59	Median: 62	77.4	69.7
Cao, 2004 [14]	CC, unmatched	China	MDR *P. aeruginosa*	*P.aeruginosa*	112	60	50	59	58.8
Cilloniz, 2016 [15]	P, cohort	Spain	MDR *P. aeruginosa*	Non-MDR *P. aeruginosa* (not fully described)	68	72.7	71.1	90.9	78.3
Cobos-Trigueros, 2015 [16]	P, cohort	Spain	MDR *P. aeruginosa*	Susceptible or resistant *P.aeruginosa* and non-*P.aeruginosa*	850	NR	NR	NR	NR
D’Agata (b), 2006 [17]	CC, matched	USA, Italy	MDR *P. aeruginosa*	Non-*P.aeruginosa*	302	58	62	62	50
Dalfino, 2011 [18]	P, cohort	NR	MDR *P. aeruginosa*	NR	251	63	63	65	61.3
Dantas, 2014 [19]	R, cohort	Brazil	MDR *P. aeruginosa*	Resistant or susceptible *P. aeruginosa*	120	51.5 (total)	51.5 (total)	63.3 (total)	63.3 (total)
Defez (a), 2004 [20]	CC, matched	France	MDR *P. aeruginosa*, nosocomial	Hospitalized, non-nosocomial MDR*P. aeruginosa*	320	73.2	67	56.25	52.5
Defez (b), 2004 [20]	CC, unmatched	France	MDR *P. aeruginosa*	Non MDR- *P. aeruginosa* (not described)	395 (155)	73.2	NR	56.25	56
Gomez-Zorilla, 2014 [21]	P, cohort	Spain	MDR *P. aeruginosa* (MDR non-XDR and XDR)	Non-MDR *P. aeruginosa* (not fully described)	112	65.3 (total)	65.3 (total)	69 (total)	69 (total)
Johnson, 2009 [22]	R, cohort	USA	MDR *P. aeruginosa*	Non-MDR *P.aeruginosa* (not described)	503	median: 59 (total)	median: 59 (total)	57 (total)	57 (total)
Joo, 2011 [23]	P, cohort	South Korea	MDR *P. aeruginosa*	Resistant or susceptible *P. aeruginosa*	202	55 (total)	55 (total)	62.9 (total)	62.9 (total)
Liew, 2013 [24]	CC, matched	Singapore	XDR *P. aeruginosa*	Non-*P. aeruginosa* or other gram-negative	79	Median: 47 (total)	NR	62 (total)	NR
Lodise, 2007 [25]	CC, unmatched	USA	MDR *P. aeruginosa*	Non-MDR *P.aeruginosa*(not fully described)^a^	351	60.5 (total)	60.5 (total)	61.2 (total)	61.2 (total)
Micek, 2015 [26]	R, cohort	USA, France, Germany, Italy, Spain	MDR *P. aeruginosa*	Non-MDR *P. aeruginosa* (not fully described)	740	53.5	62.1	62.8	70.2
Montero (a), 2010 [27]	CC, unmatched	NR	MDR *P. aeruginosa*	Non-*P. aeruginosa*	1035	67.8	67.5	72.5	54.3
Montero (b), 2010 [27]	CC, unmatched	NR	MDR *P. aeruginosa*	Susceptible *P.aeruginosa*	877	67.8	69.1	72.5	59.4
Nakamura, 2013 [28]	P, cohort	Japan	MDR *P. aeruginosa*	Non MDR *P.aeruginosa* (not fully described)^c^	435	NR	NR	NR	NR
Nseir, 2011 [29]	P, cohort	France	MDR *P. aeruginosa*	Non MDR *P. aeruginosa* (not fully described)	511	60	55	65	69
Ohmagari, 2005 [30]	CC, unmatched	USA	Patients with cancer with MDR *P. aeruginosa* infection	Patients with cancer with susceptible *P. aeruginosa* infection	54	51.8	60.3	50	58.3
Paramythiotou, 2004 [31]	CC, matched	France	MDR *P. aeruginosa*	Non-*P. aeruginosa*	68	59	61.5	65	65
Park, 2011 [32]	CC, matched	South Korea	XDR *P. aeruginosa*	Non-XDR *P.aeruginosa* (not fully described)^b^	99	65	56	79	46
Pena, 2009 [39]	P, cohort	Spain	MDR *P. aeruginosa*	CRPA	246	67	65	67	67
Pena (a), 2012 [33]	R, cohort	Spain	XDR *P. aeruginosa*	Susceptible *P. aeruginosa*	138	64.7	65.06	86	70.5
Pena (b), 2012 [33]	R, cohort	Spain	Non-XDR MDR *P. aeruginosa*	Susceptible *P. aeruginosa*	108	64.85	65.06	54	70.5
Samonis, 2014 [34]	R, cohort	Greece	XDR *P. aeruginosa*	Non-XDR *P. aeruginosa* (MDR and PDR and sensitive)	89	73	69	81.8	57.3
Tumbarello, 2011 [35]	CC, unmatched	Italy	MDR *P. aeruginosa*	Non-*P. aeruginosa*	252	62	63	57.5	57.5
Tuncer, 2012 [36]	CC, unmatched	Turkey	MDR and XDR *P. aeruginosa*	Non-MDR *P.aeruginosa* (not fully described)	120	58.6	58.2	54.1	49.4
Ustun, 2016 [37]	CC, unmatched	Turkey	MDR *P. aeruginosa*	Non-*P. aeruginosa*	225	29.8	37.9	58.7	65.3
Willmann, 2014 [38]	CC, matched	Germany	XDR *P. aeruginosa*	Non-XDR *P. aeruginosa* (not fully described) or non- *P. aeruginosa*	31	56	60	65	78.5

*CC* Case-control, *CRPA* carbapenem-resistant *P. aeruginosa*, *MDR* multi-drug resistant, *P* Parallel, *PDR* Pan-Drug-Resistant, *R* Retrospective, *XDR* extremely drug-resistant, *YR* Year

^a^Lodise 2007: Non-MDR *P.aeruginosa* = ≥90% for only amikacin, cefepime, and piperacillin-tazobactam

^b^Park,Y.S 2011:used random selection of controls

^c^Nakamura 2013:Non-MDR defines patients with P. aeruginosa other than the MDR phenotype on the same ward as those with the MDR phenotype within the same month

Of the included 28 articles, 20 articles (from 22 studies) reported data on MDR*P. aeruginosa* acquisition [12, 14–20, 22, 23, 25–31, 35, 37, 39], six reported on XDR *P. aeruginosa* [13, 24, 32–34, 38], and two included both (MDR acquisition being the most commonly reported) [21, 36]. The most commonly reported definition for MDR was laboratory-confirmed resistance to more than one agent in three or more classes of antibiotics. In five studies, the definitions were similar to the definition of XDR, but they were described as MDR [12, 16, 17, 27, 28]. The most commonly reported definition for XDR was non-susceptibility to at least one agent in all antimicrobial classes, but susceptibility to two or fewer anti-pseudomonal antimicrobials; one study required evidence of resistance to all available anti-pseudomonal antimicrobials [32].

Of 8935 patients, 2446 case patients had MDR or XDR *P. aeruginosa* or both types of resistance and the remaining were control patients. The descriptions of study control groups varied across studies: susceptible *P. aeruginosa* (5 studies [12, 13, 26, 29, 32]); non-MDR *P. aeruginosa* (without MDR phenotype) (14 studies [14, 15, 18–22, 24, 25, 27, 34, 35, 60, 39]); non-XDR *P. aeruginosa* (without XDR phenotype) (2 studies [31, 33]); non-*P. aeruginosa* (any pathogen other than *P. aeruginosa*) (4 studies [16, 23, 26, 30]); non-MDR/non-XDR *P. aeruginosa* plus non-*P. aeruginosa* (2 studies [17, 37]). No data were reported in two studies [11, 19]. The mean age of study subjects ranged from 29.8 years to 73.2 years (cases) and from 37.9 years to 71.1 years (controls). The males included as study subjects ranged from 50 to 91% (cases) and from 46 to 78.3% (controls). Most studies included a mix of patients with respiratory, wound, genitourinary, and bloodstream as the source of infection. Various severity scores were employed across studies including McCabe score, APACHE II, Charlson index, and SAPS II score.

For meta-analysis, we categorized studies based on three control groups: non-MDR *P. aeruginosa* (without MDR phenotype); susceptible *P. aeruginosa*; and any controls (non *P. aeruginosa* or undescribed controls). In analyses of risk for acquisition of MDR *P. aeruginosa*, 21 articles in 23 studies [12, 14–20, 22, 23, 25–31, 33, 35, 37, 39]) reported data on MDR *P. aeruginosa* acquisition and two included both MDR *P. aeruginosa* and XDR *P. aeruginosa* (MDR *P. aeruginosa* acquisition being the most commonly reported) [21, 36].

The risk factors statistically significantly associated with acquisition of MDR *P. aeruginosa* in meta-analysis are described in the following results sections. Additional risk factors that had a statistically significant association with acquisition of MDR *P. aeruginosa* but were not eligible for meta-analysis are listed in Additional file 1: Tables S2-S5.

#### MDR *P. aeruginosa* versus non-MDR *P. aeruginosa*

**Fig. 2:**
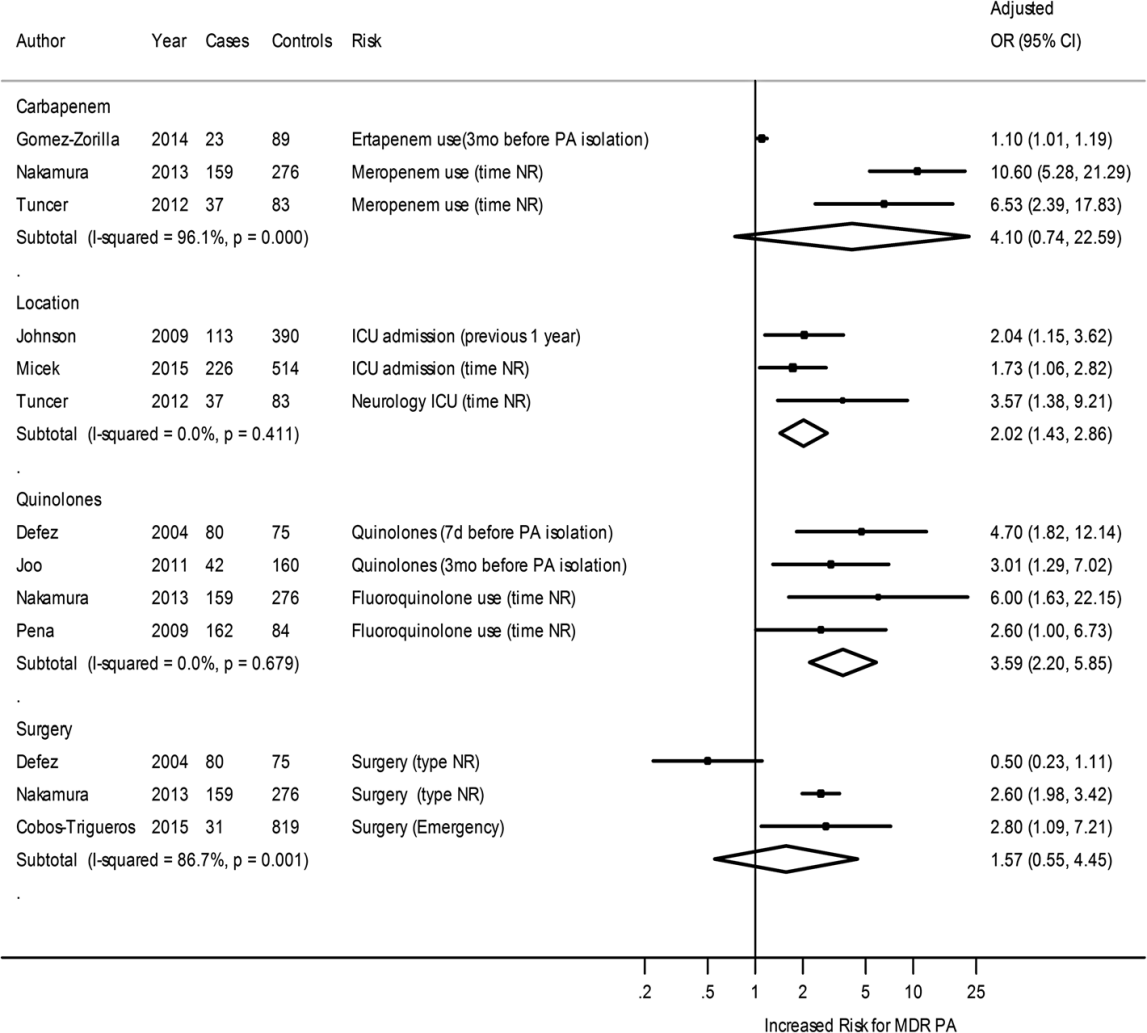
Forest plot of risk factors for MDR versus non-MDR *P. aeruginosa* acquisition. CI = Confidence Interval; ICU = Intensive Care Unit; NR = Not Reported; OR = Odds Ratio; PA = *P. aeruginosa*

Thirteen studies with non-MDR *P. aeruginosa* as a comparator reported risk factors associated with acquisition of MDR *P. aeruginosa* [15, 16, 19–23, 25, 26, 28, 35, 36, 39]. In meta-analysis, prior ICU stay or prior use of quinolones had a statistically significant association with acquisition of MDR *P. aeruginosa*. Two other predictors, surgery and prior use of carbapenems, were not associated with an increased risk of acquisition (Fig. 2).

#### MDR *P. aeruginosa* versus susceptible *P. aeruginosa*

**Fig. 3:**
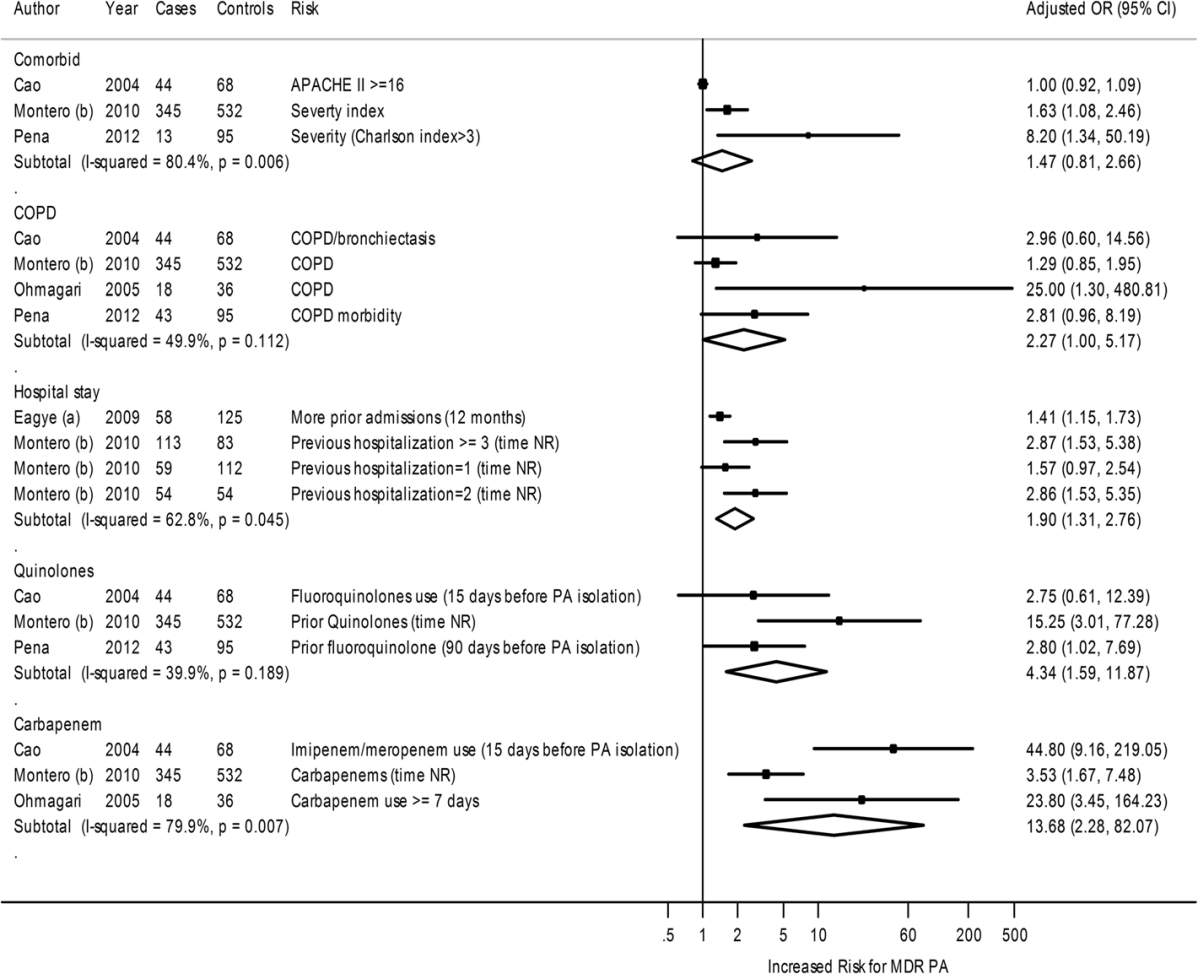
Forest plot of risk factors for MDR versus susceptible *P. aeruginosa* acquisition. APACHE II = Acute Physiology And Chronic Health Evaluation II; CI = confidence interval; COPD = Chronic obstructive pulmonary disease; NR = Not Reported; OR = Odds Ratio; PA = *P. aeruginosa*

Four studies with susceptible *P. aeruginosa* as a comparator reported risk factors for acquisition of MDR *P. aeruginosa* [14, 27, 30, 33]. In meta-analysis, prior admission, use of quinolones, and use of carbapenems had a statistically significant association with future acquisition of MDR *P. aeruginosa*. Two other predictors (comorbid severity scores and chronic obstructive pulmonary disease [COPD]) were not associated with a risk of acquisition of MDR *P. aeruginosa* in meta-analysis (Fig. 3).

Of note, the following factors were not combined in a meta-analysis; but were reported to be not associated with acquisition of MDR *P. aeruginosa*: colonization of *P. aeruginosa* in isolate sources other than blood preceding a diagnosis of bacteremia, isolation of multiple pathogens in culture, and history of previous *P. aeruginosa* infection.

#### MDR *P. aeruginosa* versus non-*P. aeruginosa*

**Fig. 4:**
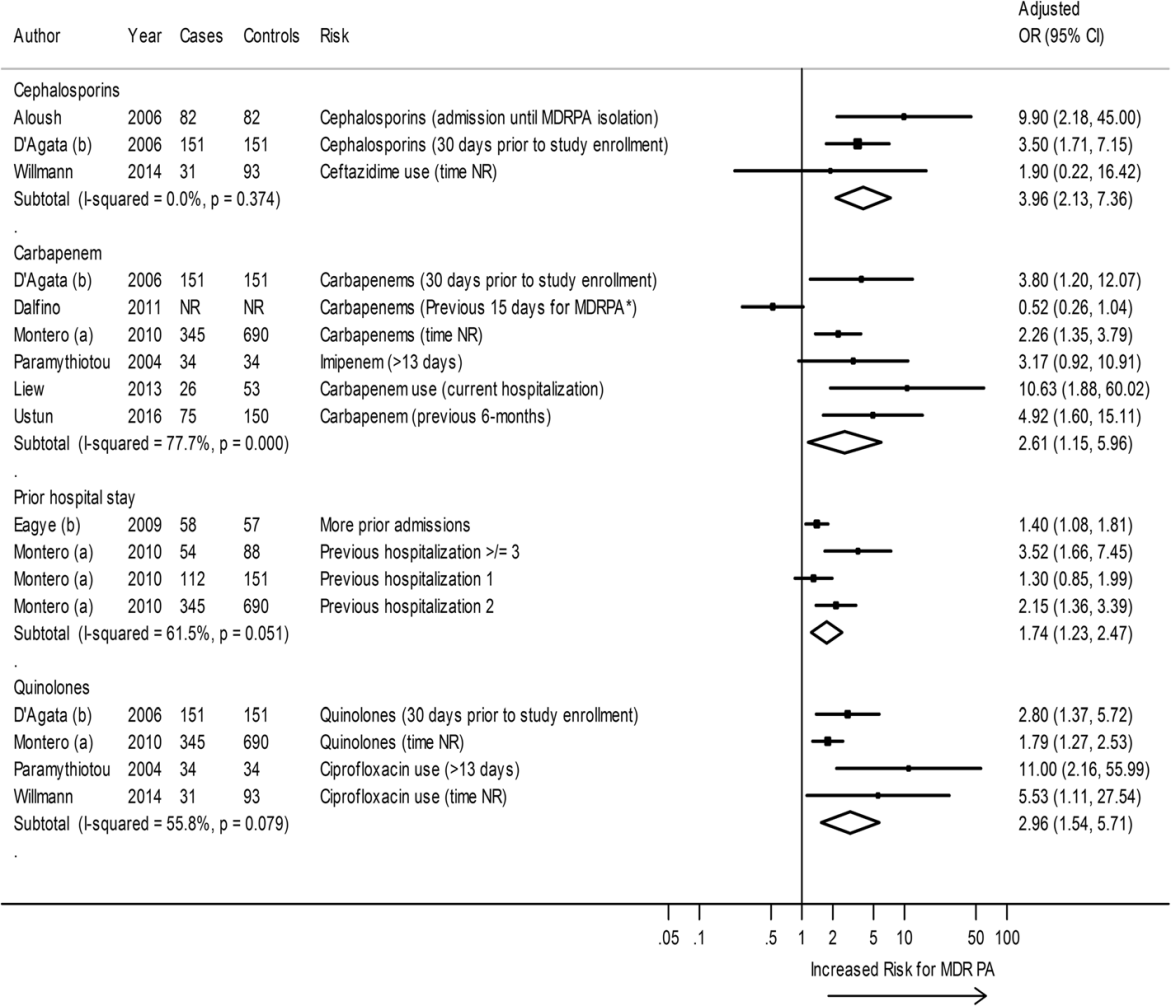
Forest plot of risk factors for MDR versus Non-*P. aeruginosa* acquisition. *Dalfino 2011, ICU stay was used for the control. CI = confidence interval; MDRPA = multi-drug resistant *P. aeruginosa*; NR = not reported; OR = Odds Ratio

Seven studies with non-*P. aeruginosa* or any undescribed control as a comparator reported risk factors for acquisition of MDR *P. aeruginosa* [12, 17, 18, 27, 29, 31, 37]. In meta-analysis, penicillins, carbapenems, quinolones, disease severity, and prior admissions predicted a statistically significant increased risk of acquiring MDR *P. aeruginosa*, compared with non-*P. aeruginosa* (Fig. 4).

Both diabetes and sepsis were not associated with the risk of acquiring MDR *P. aeruginosa*, compared with non-*P. aeruginosa*.

#### XDR *P. aeruginosa* studies

Six studies reported risk factors for acquisition of XDR *P. aeruginosa* alone, compared with different controls [13, 24, 32–34, 38]. XDR *P. aeruginosa* was compared with non-XDR *P. aeruginosa* (2 studies [32, 34]); non-XDR *P. aeruginosa* and non-*P. aeruginosa* (1 study [38]); non-*P. aeruginosa* (1 study [24]); susceptible *P. aeruginosa* (1 study [33]); and susceptible *P. aeruginosa* and non-*P. aeruginosa* (1 study [13]).The most frequently reported factors that significantly increased the risk of acquiring XDR *P. aeruginosa* included: prior fluoroquinolones use (2 studies), urinary catheter (2 studies). The following factors were not associated with an increased risk of XDR *P. aeruginosa*: ceftazidime use, days of ciprofloxacin use, number of different antibiotics used during time at risk (i.e. time between admission and isolation of bacteria in index cases).

### Studies of resistant *P. aeruginosa*

#### Carbapenem-resistant *P. aeruginosa*

Twenty-three studies in 21 articles (*n* = 9877 subjects) that examined risk factors for acquisition of carbapenem-resistant *P. aeruginosa* [17, 19, 40, 42–47, 50, 51, 54–57, 59, 60, 61, 62, 64, 65]; two articles contributed to two studies each [44, 64]. Five were prospective studies and the remaining 16 had case-control or retrospective designs. The studies were conducted in Brazil (8 studies), the USA (5 studies), Western Europe (4 studies), Asia (3 studies), and the USA and Western Europe (1 study). All studies were conducted in tertiary care or university hospitals. Three were multicenter studies. Table 2 summarizes baseline characteristics of included carbapenem resistant *P. aeruginosa* studies.

**Table 2:** Baseline characteristics of studies in carbapenem-resistant *P. aeruginosa*

Author	Year	Design	Country	Case/Exposure	Control/Comparator	Total N	Age cases (Yr)	Age control (Yr)	Male cases (%)	Male control (%)
D’Agata (a) [17]	2006	CC, matched	USA, Italy	CRPA	Non-*P. aeruginosa*	82	61	60	73	44
DalBen [42]	2013	P, cohort	Brazil	MRAC and CRPA	Non-MRAC and non-CRPA	325	44	41	59	41
Djordjevic [43]	2013	P, cohort	Serbia	CRPA	CSPA	261	59.2	61.4	80.8	64.9
Eagye (a) [44]	2009	CC, control	USA	MRPA	MSPA	183	66.4	66.1	58.6	59.2
Eagye (b) [44]	2009	CC, control	USA	MRPA	Non-*P. aeruginosa*	182	66.4	57.4	58.6	50.9
Fortaleza (a) [45]	2006	CC, matched	Brazil	IRPA	Control (NR)	324	median 45	median 44	68.5	60.2
Furtado [46]	2009	CC, matched	Brazil	IRPA	Without IRPA	245	50	54	68.3	59.3
Furtado [47]	2010	CC, matched	Brazil	IRPA	Non-IRPA	295	54	54	70.7	56.5
Harris [50]	2002	CC, matched	USA	IRPA	Without IRPA	866	55.7	49.4	39.1	39.1
Harris [51]	2011	P, cohort	USA	IRPA	Non-IRPA	3146	56.7	55.7	61.9	56.7
Kohlenberg [54]	2010	CC, unmatched	Germany	CRPA	CSPA	33	median 60	median 44.4	53.3	72.2
Lautenbach [55]	2010	CC, unmatched	USA	IRPA	ISPA	2542	61 (total)	61 (total)	63.3	56.1
Lee [56]	2015	CC, matched	Taiwan	CRPA	All susceptible *P. aeruginosa*	75	61.6	62	48	48
Lin [57]	2016	CC, unmatched	Taiwan	CRPA	CSPA	164	66.6	63.5	72	67.1
Luyt [58]	2014	P, cohort	France	CRPA	CSPA	169	57.6	57.9	65	66
Onguru [59]	2008	P, cohort	Turkey	IRPA	ISPA	170	45.9	49.7	72	66.3
Pena [60]	2007	P, cohort	Spain	CRPA	CSPA	254	59.8	57.1	60	65
Pereira [61]	2008	CC, unmatched	Brazil	IRPA	ISPA	59	51.8	50.7	70	55
Tam [62]	2007	CC, unmatched	USA	CRPA	Pan-susceptible *P. aeruginosa*	51	50	64	33	73
Tuon [40]	2012	CC, unmatched	Brazil	CRPA	CSPA	77	46.4	49	75.9	70.8
Zavascki (a) [64]	2005	CC, unmatched	Brazil	IRPA	Non *P. aeruginosa*	186	58	51	60	53
Zavascki (b) [64]	2005	CC, unmatched	Brazil	IRPA	ISPA	158	58	51	60	66
Zhang [65]	2009	CC, unmatched	China	CRPA	CSPA	34	61	50	NR	NR

*CC* case-control, *CRPA* carbapenem-resistant*P. aeruginosa*, *CSPA* carbapenem-susceptible *P.aeruginosa*, *ICU* Intensive Care Unit, *IRPA* imipenem-resistant *P. aeruginosa*, *ISPA* imipenem-susceptible *P.aeruginosa*, *MRAC* meropenem-resistant *Acinetobacter baumannii*, *MRPA* meropenem-resistant *P. aeruginosa*, *MSPA* meropenem-susceptible *P. aeruginosa*, *NR* not reported,k *P* parallel, *SICU* Surgical Intensive Care Unit, *YR* year

**Table 3:** Baseline characteristics of studies in resistant *P. aeruginosa*

Author	Year	Design	Country	Case/Exposure	Control/Comparator	Total N	Age cases (Yr)	Age control (Yr)	Male cases (%)	Male controls (%)
Akhabue [41]	2011	CC, unmatched	USA	Cefepime-resistant *P.aeruginosa*	Cefepime-susceptible *P.aeruginosa*	2529	Median:61 (total)	Median:61 (total)	62	56.4
Fortaleza (b) [45]	2006	CC, matched	Brazil	Ceftazidime-resistant *P.aeruginosa*	Control	165	median 38	median 44.5	72.7	62.7
Gasink [48]	2006	CC, unmatched	USA	Fluoroquinolone-resistant *P.aeruginosa*	Fluoroquinolone-susceptible *P.aeruginosa*	847	56	62	NR	NR
Harris [49]	2002	CC, unmatched	USA	Piperacillin-tazobactam-resistant *P. aeruginosa*	Patients without Piperacillin-tazobactam-resistant *P. aeruginosa*	1315	53.4	49.7	42.5	39.4
Hsu [52]	2005	CC, unmatched	USA	Fluoroquinolone-resistant *P.aeruginosa*	Fluoroquinolone-susceptible *P.aeruginosa*	177	73.8	68	43	49
Khayr [53]	2000	CC, unmatched	USA	Ciprofloxacin-resistant *P.aeruginosa*	Ciprofloxacin-susceptible *P.aeruginosa*	94	Age > 65: 79%	Age > 65: 79%	100	100
Trouillet [63]	2002	CC, unmatched	France	Piperacillin-resistant *P. aeruginosa*	Piperacillin-susceptible *P. aeruginosa*	135	64.6	65.5	67.6	64.4

*CC* case-control, *NR* not reported, *YR* year

The descriptions of control groups varied across studies. For the purpose of analysis, we categorized the controls into two categories: susceptible *P. aeruginosa* and any control (non-*P. aeruginosa* with or without susceptible *P. aeruginosa*). The mean age ranged from 44 years to 67 years (cases) and from 41 years to 66 years (controls). Most studies included a mix of patients with respiratory, wound, genitourinary, and bloodstream as the source of infection.

#### Carbapenem-resistant versus susceptible *P. aeruginosa*

In meta-analysis, prior use of piperacillin-tazobactam, vancomycin, and carbapenems were all significantly associated with acquisition of carbapenem-resistant *P. aeruginosa* (Additional file 1: Figures S1-S2). Other predictors including use of quinolones, hospital stay, and time at risk were not associated with a statistically significant increased risk (Additional file 1: Figures S1-S2). Additional risk factors that significantly increased the risk of acquiring carbapenem-resistant *P. aeruginosa* are summarized in Additional file 1: Tables S6-S9.

#### Carbapenem-resistant versus any control (unspecified or non- *P. aeruginosa* controls)

Four studies used other controls (3 studies that used Non-P.aeruginosa controls and one study did not specify the control) [17, 44, 45, 64]. No meta-analysis could be conducted as there were fewer than three studies for this comparison for the same type of predictor. All studies reported a statistically significant increased risk of carbapenem-resistant *P. aeruginosa*, compared with any control. The risk factors examined included prior use of antibiotics, comorbid score, length of hospital or ICU stay, hemodialysis, non-ambulatory status, transfer from another facility, indwelling urinary catheter, and mechanical ventilation.

### Other (non-carbapenem) resistant *P. aeruginosa*

Of the seven studies that reported data on acquisition of other (non-carbapenem) resistant *P. aeruginosa* in Table 3 [41, 45, 48, 49, 52, 53, 63], two reported data on piperacillin- or piperacillin-tazobactam-resistant *P. aeruginosa* [49, 63], three reported data on quinolone-resistant *P. aeruginosa* [48, 52, 53], and two reported on *P. aeruginosa* resistant to the newer cephalosporins, namely, cefepime and ceftazidime [41, 45].

#### Piperacillin- or piperacillin-tazobactam-resistant *P. aeruginosa*

No meta-analysis could be conducted as there were fewer than three studies for this comparison on any particular category of predictor [49, 63]. Two studies reported a statistically significant increased risk for piperacillin- or piperacillin-tazobactam*-*resistant *P. aeruginosa* for the following factors: admissions in prior year, ICU stay, time at risk, transfer, severe morbidity or higher comorbid score, and prior use of antibiotics (aminoglycosides, broad-spectrum cephalosporins, carbapenems, piperacillin-tazobactam, and quinolones).

#### Quinolone-resistant *P. aeruginosa*

Three studies compared quinolone-resistant and susceptible *P. aeruginosa* [48, 53, 53]. In meta-analysis, prior use of quinolones predicted subsequent risk for acquisition of quinolone-resistant *P. aeruginosa* (Additional file 1: Figure S3)*.* Other statistically significant risk factors for an increased acquisition of quinolone-resistant *P. aeruginosa* included indwelling airway, co-existing diabetes mellitus, and nosocomial residence (not defined) (Additional file 1: Table S10). The number of hospital days from admission to culture was not a significant predictor.

#### Third-generation cephalosporin-resistant *P. aeruginosa*

Two studies [41, 45] reported an increased risk for third-generation cephalosporin*-*resistant *P. aeruginosa* for the following factors: transfer from outside facility, prior use of antibiotics (e.g. extended-spectrum cephalosporins, extended-spectrum penicillin, or quinolones or amikacin). Both length of hospital stay before culture and prior use of carbapenem were not associated with an increased risk for resistance to higher generation cephalosporins in one study [41].

## Discussion

This systematic review of the literature summarizes risk factors that predict acquisition of MDR *P. aeruginosa*, XDR *P. aeruginosa*, and other resistant *P. aeruginosa*. Our meta-analysis identified that the risk factors for acquisition of MDR or XDR *P. aeruginosa* varied with regard to the type of control used for comparison. ICU stays (either as current or prior to current episode of infection) and use of quinolones was significantly associated with acquisition of MDR or XDR *P. aeruginosa*, compared with resistant or susceptible *P. aeruginosa*. Use of prior antibiotics including, cephalosporins, quinolones, or carbapenems, and prior hospital admissions predicted an increased risk of MDR or XDR *P. aeruginosa* versus non-*P. aeruginosa*.

The increasing rates of MDR or XDR, or resistant *P. aeruginosa* are a worldwide public health problem. Resistant strains of *P. aeruginosa* are associated with high mortality and increased resource utilization [66]. The emergence and spread of MDR *P. aeruginosa* may be associated with misuse or overuse of antimicrobials. Our meta-analysis found a consistent association between the use of quinolones and acquisition of MDR or XDR *P. aeruginosa P. aeruginosa*. However, association between carbapenem use and acquisition of MDR or XDR *P. aeruginosa* was not consistent across comparisons. While some primary studies did report a relationship between MDR or XDR *P. aeruginosa* and prior use of antibiotics [14, 30], our meta-analysis identified statistically significant associations between carbapenem use and acquisition of MDR or XDR *P. aeruginosa* and the significance of their association can vary by the type of comparator examined. Nonetheless, our results confirm that prior use of specific antimicrobials is an important risk factor for acquisition of MDR or XDR *P. aeruginosa*. Antimicrobial resistance occurs over time, often mediated by gene mutations and misuse or overuse of antimicrobials may accelerate this process. Understanding the epidemiology of MDR or XDR *P. aeruginosa* will be necessary to overcome infections with these resistant pathogens.

This review finding can be supported in the context of a recent review on MDR or XDR *P. aeruginosa*, which examined multivariate risk factors reported in eight included articles [67]. The authors of this review did not perform a meta-analysis but identified prior antimicrobial therapy, medical devices, patient-related characteristics, and environmental sources as risk factors for MDR or XDR *P. aeruginosa*. Our review, in which a meta-analysis was performed, demonstrated that admission location, prior admission, or use of quinolones was significantly associated with acquisition of MDR or XDR *P. aeruginosa*. In addition to reviewing MDR or XDR *P. aeruginosa*, we reviewed single drug-resistant *P. aeruginosa.* In meta-analysis, prior use of piperacillin-tazobactam, vancomycin, or carbapenems were significantly associated with acquisition of carbapenem-resistant versus susceptible *P. aeruginosa*. In meta-analysis, prior fluoroquinolone use was a statistically significant predictor of subsequent quinolone-resistant versus susceptible *P. aeruginosa*.

There are limitations associated with this systematic literature review. To begin with, the study inclusion criteria were limited to published studies indexed in English. Secondly, studies eligible for inclusion were heterogeneous with respect to the definition of exposure, site of infection with *P. aeruginosa,* and risk factors included in the study. As a result, a combined meta-analysis may misrepresent the true picture. We mitigated these issues by using a random effects model in our analyses and by limiting analyses to similar comparators. However, in many instances there were risk factors that were statistically significant in two studies, but unfortunately a third study was not available to permit the exploration of this factor through meta-analysis. Additionally, our study results may not be generalizable to all regions as studies from certain regions (for example, Western European nations for MDR or XDR *P. aeruginosa* and Brazil for carbapenem-resistant *P. aeruginosa*) were overrepresented in our sample published literature.

Confounding in observational studies reporting unadjusted data is a well-known source of bias. To mitigate this bias, we included risk factors that were analyzed in multivariate analyses. The choice of the control group (e.g. patients with infection with susceptible strains or patients without any infection at all) may also have influenced the results. We attempted to address this issue using similar comparison groups in the meta-analyses. As with any evidence synthesis, the limitations of the data available in primary studies will transfer into limitations of the systematic review. For example, the control group was not well defined in studies, specifically in studies that reported non- *P. aeruginosa* as controls. The description of these controls was unclear if the control group did or did not include MDR infection of a non-Pseudomonas bacteria. The results section describes variability across studies with regard to study characteristics, outcome assessment, and the relationship between risk factors and the outcome of acquisition of resistant *P. aeruginosa*. We could not conduct additional subgroup analyses by site of infection, or explain differences across studies using stratified analyses due to the small number of available studies for any particular comparison. An insufficient number of studies (< 10 studies) precluded evaluatingthe potential for publication bias with funnel plots and Egger’s tests for small study effects [68].

## Conclusions

Consistently, across comparisons, prior use of antibiotics and prior ICU stay was a significant risk predictor for acquisition of MDR or XDR *P. aeruginosa* infections. Depending on local epidemiology, this finding is useful in identifying patients at high risk for resistant *P. aeruginosa* that may benefit from alternate empiric treatment. Further, these findings emphasize the need for antimicrobial stewardship and infection control in hospitals and continued need for the development of new antimicrobial agents with activity against MDR *P. aeruginosa* [68]. The implementation of antimicrobial stewardship and infection control in hospitals can improve patient safety and care, reduce resource utilization, and reduce resistance. The increasing prevalence of antimicrobial resistance among hospitalized patients continues to pose a challenge for practitioners.

## Additional file

Additional file 1: **Table S1.** Search Strategy. **Table S2.** Patient–related Multivariate Risk Factors of Acquistion of MDR *P. aeruginosa*. **Table S3.** Antibiotic Treatment–related Multivariate Risk Factors of Acquistion of MDR *P. aeruginosa*. **Table S4.** Other Treatment–related Multivariate Risk Factors of Acquistion of MDR and XDR *P. aeruginosa*. **Table S5.** Hospital–related Multivariate Risk Factors of Acquistion of MDR and XDR *P. aeruginosa*. **Table S6.** Patient–related Multivariate Risk Factors of Acquistion of Carbapenem-resistant *P. aeruginosa*. **Table S7.** Antibiotic Treatment–related Multivariate Risk Factors of Acquistion of Carbapenem-resistant *P. aeruginosa*. **Table S8**. Other Treatment–related Multivariate Risk Factors of Acquistion of Carbapenem-resistant *P. aeruginosa*. **Table S9.** Hospital–related Multivariate Risk Factors of Acquistion of Carbapenem-resistant *P. aeruginosa*. **Table S10.** Multivariate Risk Factors of Acquistion of Resistant *P. aeruginosa*. **Figure S1.** Meta-analysis of Risk Factors for Carbapenem versus Susceptible *P. aeruginosa* Acquisition. **Figure S2.** Meta-analysis of Prior Use of Carbapenem as a Risk Factor for Carbapenem versus Susceptible *P. aeruginosa* Acquisition. **Figure S3.** Meta-analysis of Prior Use of Fluoroquinolones as a Risk Factor for Quinolone-resistant versus Susceptible *P. aeruginosa* Acquisition. (DOCX 182 kb)

## Abbreviations

AHRQ: Agency for Healthcare Research and Quality
APACHE II: Acute Physiology and Chronic Health Evaluation II
COPD: Chronic obstructive pulmonary disease
ESBL: Extended-spectrum beta-lactamase organisms
HAI: Hospital acquired infection
ICU: Intensive care unit
MDR: Multi-drug resistant
OR: Odds ratio
SAPS II: Simplified acute physiology score II
US: United States
XDR: Extensively drug resistant
