# Supplementary material for: Risk factors for hospitalized patients with resistant or multidrug-resistant Pseudomonas aeruginosa infections: a systematic review and meta-analysis
Source: Antimicrob Resist Infect Control. 2018 Jul 4;7:79. doi: 10.1186/s13756-018-0370-9 (PMC6032536; doi:10.1186/s13756-018-0370-9)
Supplement: Supplementary file 1 — Table S1. Search Strategy. Table S2. Patient–related Multivariate Risk Factors of Acquistion of MDR P. aeruginosa. Table S3. Antibiotic Treatment–related Multivariate Risk Factors of Acquistion of MDR P. aeruginosa. Table S4. Other Treatment–related Multivariate Risk Factors of Acquistion of MDR and XDR P. aeruginosa. Table S5. Hospital–related Multivariate Risk Factors of Acquistion of MDR and XDR P. aeruginosa. Table S6. Patient–related Multivariate Risk Factors of Acquistion of Carbapenem-resistant P. aeruginosa. Table S7. Antibiotic Treatment–related Multivariate Risk Factors of Acquistion of Carbapenem-resistant P. aeruginosa. Table S8. Other Treatment–related Multivariate Risk Factors of Acquistion of Carbapenem-resistant P. aeruginosa. Table S9. Hospital–related Multivariate Risk Factors of Acquistion of Carbapenem-resistant P. aeruginosa. Table S10. Multivariate Risk Factors of Acquistion of Resistant P. aeruginosa. Figure S1. Meta-analysis of Risk Factors for Carbapenem versus Susceptible P. aeruginosa Acquisition. Figure S2. Meta-analysis of Prior Use of Carbapenem as a Risk Factor for Carbapenem versus Susceptible P. aeruginosa Acquisition. Figure S3. Meta-analysis of Prior Use of Fluoroquinolones as a Risk Factor for Quinolone-resistant versus Susceptible P. aeruginosa Acquisition. (DOCX 182 kb) [file 13756_2018_370_MOESM1_ESM.docx]

Table S1. Search Strategy

| **#** | **Searches** |
| --- | --- |
| 1 | *Pseudomonas Infections/mi or *Pseudomonas Infections/ep or Pseudomonas Infections/di |
| 2 | Gram-Negative Bacterial Infections/di |
| 3 | 1 or 2 |
| 4 | (beta-Lactamases or metallo-beta-lactamases or MBL or MBLs).tw. |
| 5 | 3 and 4 |
| 6 | exp Drug Resistance, Bacterial/ or *Drug Resistance, Bacterial/ |
| 7 | exp Drug Resistance multiple, Bacterial/ or *Drug Resistance, Multiple, Bacterial/ |
| 8 | (resistant or resistance or MDR or multidrug resistance or multidrug resistant or multi-drug resistance or multi-drug resistant or multidrug-resistant or multidrug-resistance or extreme-drug resistant or XDR or extensive drug-resistant or multiresistant or Carbapenem-resistant or aztreonam-resistant).tw. |
| 9 | or/6-8 |
| 10 | 3 and 9 |
| 11 | (MDR-PA or XDRPA or CRPA or MR-PA).tw. |
| 12 | 5 or 10 or 11 |
| 13 | (nosocomial or hospital-acquired or healthcare-acquired or hospital-associated or healthcare-associated or hospital or hospitals or hospitalized or (intensive and care) or (critical and care)).af. |
| 14 | *Cross Infection/ep or *Cross Infection/mi |
| 15 | exp Emergency service, hospital/ |
| 16 | exp hospitals/ |
| 17 | or/13-16 |
| 18 | 12 and 17 |
| 19 | predictive value of tests/ |
| 20 | risk assessment/ |
| 21 | multivariate analysis/ |
| 22 | risk factors/ |
| 23 | logistic models/ |
| 24 | predict:.tw. |
| 25 | (risk and scor:).tw. |
| 26 | scor:.tw. |
| 27 | validat:.tw. |
| 28 | or/19-27 |
| 29 | 18 and 28 |
| 30 | limit 29 to yr="2000 -Current" |

Table S2. Patient –related Multivariate Risk Factors of Acquistion of MDR P. aeruginosa

| Author | Year | N Cases / Exposure | N comparator | Risk Factors /Predictors | Multivariate metric | Results | 95%LCI | 95%UCI | P-value |
| --- | --- | --- | --- | --- | --- | --- | --- | --- | --- |
| Age |  |  |  |  |  |  |  |  |  |
| Cao | 2004 | 44 | 68 | Age>=60 years | Adjusted OR | 10.01 | 0.98 | 1.05 | 0.374 |
| Defez | 2004 | 17 | 67 | Age 54–69 | Adjusted OR | 0.72 | 0.2 | 2.9 | 0.62 |
| Defez | 2004 | 21 | 51 | Age 70–78 | Adjusted OR | 2.7 | 0.7 | 9.9 | 0.12 |
| Defez | 2004 | 31 | 52 | Age 79–98 | Adjusted OR | 4.5 | 1.3 | 16.5 | 0.02 |
| Gomez-Zorilla | 2014 | 23 | 89 | Age >65 yr | Adjusted HR | 1.2 | 0.47 | 3.04 | 0.69 |
| Eagye (b) | 2009 | 58 | 57 | Increased age (5-year increments) | Adjusted OR | 1.17 | 1.03 | 1.33 | 0.017 |
| Micek | 2015 | 226 | 514 | Age (decreasing increments of 1) | Adjusted OR | 0.97 | 0.96 | 0.98 | <0.001 |
| Sex |  |  |  |  |  |  |  |  |  |
| Montero (a) | 2010 | 345 | 690 NON PA | Sex (Male) | Adjusted OR | 1.67 | *1.178* | *2.368* | 0.004 |
| Montero (b) | 2010 | 345 | 532 | Sex (Male) | Adjusted OR | 1.61 | *1.093* | *2.372* | 0.016 |
| Gomez-Zorilla | 2014 | 23 | 89 | Sex (Male) | Adjusted HR | 0.43 | 0.12 | 1.53 | 0.19 |
| Cobos-Trigueros | 2015 | 31 | 819 | Sex (Male) | Adjusted OR | 3.1 | 1.2 | 7.8 | 0.018 |
| Severity Score |  |  |  |  |  |  |  |  |  |
| Cao | 2004 | 44 | 68 | APACHE II >=16 | Adjusted OR | 1.001 | 0.916 | 1.095 | 0.977 |
| D'Agata (b) | 2006 | 151 | 151 | Charlson score of >2 | Adjusted OR | 3.3 | 1.8 | 6 | <0.001 |
| Pena | 2012 | 13 | 95 | Severity (Charlson index>3) | Adjusted OR | 8.2 | 1.34 | 50.2 | 0.02 |
| Gomez-Zorilla | 2014 | 23 | 89 | Charlson index of >=3 | Adjusted HR | 2.05 | 0.76 | 5.52 | 0.15 |
| Defez | 2004 | 31 | 38 | MacCabe score 1 vs. 0 | Adjusted OR | 2.9 | 1.1 | 7.8 | 0.03 |
| Defez | 2004 | 12 | 20 | MacCabe score 2 vs. 0 | Adjusted OR | 1.4 | 0.3 | 5.8 | 0.61 |
| Park,Y.S | 2011 | 33 | 66 | APACHE II score | Adjusted OR | 1.2 | 1 | 1.3 | 0.007 |
| Montero (a) | 2010 | 345 | 690 | Severity index (4 versus 1-3) | Adjusted OR | 4.29 | *1.802* | *10.214* | <0.001 |
| Montero (b) | 2010 | 345 | 532 | Severity index (4 versus 1-3) | Adjusted OR | 1.63 | *1.080* | *2.460* | 0.02 |
| Severity of Illness |  |  |  |  |  |  |  |  |  |
| Defez | 2004 | 80 | 240 | Bedridden | Adjusted OR | 9.4 | 1 | 84.1 | 0.04 |
| Aloush | 2006 | 82 | 82 | Being bedridden | Adjusted OR | 3.5 | *1.059* | *11.569* | 0.04 |
| Co-conditions |  |  |  |  |  |  |  |  |  |
| COPD |  |  |  |  |  |  |  |  |  |
| Cao | 2004 | 44 | 68 | COPD/bronchiectasis | Adjusted OR | 2.96 | 0.602 | 14.56 | 0.182 |
| Ohmagari | 2005 | 18 | 36 | COPD | Adjusted OR | 25 | 1.3 | 480.9 | 0.033 |
| Montero (a) | 2010 | 345 | 690 (Non-PA) | COPD | Adjusted OR | 2.02 | *1.329* | *3.071* | <0.001 |
| Montero (b) | 2010 | 345 | 532 (SPA) | COPD | Adjusted OR | 1.29 | *0.85* | *1.95* | 0.226 |
| Pena (XDR PA) | 2012 | 43 | 95 | COPD | Adjusted OR | 2.81 | 0.96 | 8.15 | 0.057 |
| Malignancy |  |  |  |  |  |  |  |  |  |
| Aloush | 2006 | 82 | 82 | Malignant disease | Adjusted OR | 0.2 | *0.047* | *0.856* | 0.03 |
| Samonis | 2014 | 22 (episodes) | 75 (episodes) | hematologic malignancy | Adjusted OR | 40.7 | 4.5 | 367.6 | NR |
| Ustun | 2016 | 75 | 150 | Malignant disease | Adjusted RR | 12.5 | 2.64 | 59.2 | 0.001 |
| Diabetes |  |  |  |  |  |  |  |  |  |
| Nakamura | 2013 | 159 | 276 | Diabetes mellitus | Adjusted OR | 4.0 | 2.4 | 6.7 | <0.001 |
| Micek | 2015 | 226 | 514 | Diabetes mellitus | Adjusted OR | 1.9 | 1.21 | 3 | 0.006 |
| Ustun | 2016 | 75 | 150 | Diabetes mellitus | Adjusted RR | 5.21 | 0.98 | 27.71 | 0.53 |
| Others |  |  |  |  |  |  |  |  |  |
| Eagye (a) | 2009 | 58 | 125 | Congestive heart failure | Adjusted OR | 2.19 | 1.03 | 4.68 | 0.043 |
| Ustun | 2016 | 75 | 150 | Burn | Adjusted RR | 13.6 | 4.07 | 45.8 | <0.001 |
| Ustun | 2016 | 75 | 150 | Pneumonia | Adjusted RR | 11.91 | 2.44 | 58.16 | 0.002 |
| Bodro | 2015 | 31 strains (24 patients) | 318 | Septic shock | Adjusted OR | 3.3 | 1.4 | 7.7 | 0.006 |
| Ustun | 2016 | 75 | 150 | Bacteremia/sepsis | Adjusted RR | 4.67 | 0.93 | 23.48 | 0.61 |
| Samonis | 2014 | 22 (episodes) | 75 (episodes) | Lymphopenia | Adjusted OR | 0.16 | 0.03 | 0.92 | NR |

COPD = Chronic obstructive pulmonary disease; HR = Hazard ratio; OR = odds ratio

Italicized CIs indicate values were calculated using estimate and P-value

Table S3. Antibiotic Treatment–related Multivariate Risk Factors of Acquistion of MDR P. aeruginosa

| Author | Year | N  Cases / Exposure | N comparator | Risk Factors /Predictors | Multivariate metric | Results | 95% LCI | 95% UCI | P-value |
| --- | --- | --- | --- | --- | --- | --- | --- | --- | --- |
| Quinolones |  |  |  |  |  |  |  |  |  |
| Cao | 2004 | 44 | 68 | Fluoroquinolones (15 days before PA isolation) | Adjusted OR | 2.749 | 0.61 | 12.4 | 0.188 |
| Defez | 2004 | 80 | 75 (Infected with P. aeruginosa) | Fluoroquinolones (7 days before PA isolation) | Adjusted OR | 4.7 | 1.8 | 12 | 0.001 |
| Defez | 2004 | 80 | 240 (hospitalized patients) | Fluoroquinolones (7 days before PA isolation) | Adjusted OR | 4.1 | 1.5 | 11.7 | 0.007 |
| Paramythiotou | 2004 | 34 | 34 | Ciprofloxacin (>13 days use) | Adjusted OR | 11 | 1.27 | 32.9 | NR |
| D'Agata (b) | 2006 | 151 | 151 | Quinolones (30 days prior to enrollment) | Adjusted OR | 2.8 | 1.2 | 5 | 0.001 |
| Pena | 2009 | 162 | 84 | Fluoroquinolones (time NR) | Adjusted OR | 2.6 | 1 | 6.7 | NR |
| Montero (a) | 2010 | 345 | 690 NON PA | Quinolones (time NR) | Adjusted OR | 1.79 | *1.27* | *2.53* | 0.001 |
| Montero (b) | 2010 | 345 | 532 SPA | Quinolones (time NR) | Adjusted OR | 15.25 | *3.01* | *77.28* | <0.001 |
| Nakamura | 2013 | 159 | 276 | Fluoroquinolones (time NR) | Adjusted OR | 6 | 1.6 | 21.8 | 0.007 |
| Joo | 2011 | 42 | 160 | Fluoroquinolones (3 months before PA isolation) | Adjusted OR | 3.01 | 1.29 | 7.01 | 0.011 |
| Pena (XDR) | 2012 | 43 | 95 SPA | Fluoroquinolones (90 days before PA isolation) | Adjusted OR | 2.8 | 1.02 | 7.7 | 0.04 |
| Samonis (XDR) | 2014 | 22 (episodes) | 75 (episodes) | Fluoroquinolones (time NR) | Adjusted OR | 11 | 2 | 60.5 | NR |
| Willmann (XDR) | 2014 | 31 | 93 | Ciprofloxacin (time NR) | Adjusted OR | 5.53 | 1.11 | 27.53 | 0.025 |
| Willmann (XDR) | 2014 | 31 | 93 | Ciprofloxacin, antibiotic-days, per unit increase | Adjusted OR | 1.01 | 0.88 | 1.16 | 0.88 |
| Willmann (XDR) | 2014 | 31 | 93 | Ciprofloxacin total dose, Defined Daily Dose | Adjusted OR | 1.01 | 0.87 | 1.17 | 0.91 |
| Carbapenem |  |  |  |  |  |  |  |  |  |
| Cao | 2004 | 44 | 68 | Imipenem/meropenem (15 days before PA isolation) | Adjusted OR | 44.8 | 9.16 | 219 | <0.001 |
| Paramythiotou | 2004 | 34 | 34 | Imipenem (>13 days use) | Adjusted OR | 3.17 | 0.92 | 10.9 | NR |
| Ohmagari | 2005 | 18 | 36 | Imipenem or meropenem use >/= 7 defined daily dose | Adjusted OR | 23.8 | 3.5 | 166.67 | 0.001 |
| D'Agata (b) | 2006 | 151 | 151 | Imipenem (30 days prior to enrollment) | Adjusted OR | 3.8 | 1.2 | 12.1 | 0.02 |
| Montero (a) | 2010 | 345 | 690 NON PA | *Carbapenems not specified* (time NR) | Adjusted OR | 2.26 | *1.35* | *3.79* | 0.002 |
| Montero (b) | 2010 | 345 | 532 | *Carbapenems not specified* (time NR) | Adjusted OR | 3.53 | *1.67* | *7.48* | <0.001 |
| Dalfino | 2011 | NR | NR | *Carbapenems not specified* (< 2 previous weeks)* | Adjusted OR | 0.52 | 0.2 | 0.8 | 0.0047 |
| Nakamura | 2013 | 159 | 276 | Meropenem use (time NR) | Adjusted OR | 10.6 | 5.6 | 22.6 | <0.001 |
| Gomez-Zorilla | 2014 | 23 | 89 | Ertapenem (3 mo before PA isolation) | Adjusted HR | 1.1 | 1.01 | 1.19 | 0.026 |
| Tuncer | 2012 | 37 | 83 | Meropenem (time NR) | Adjusted OR | 6.53 | 2.39 | 17.82 | <0.0001 |
| Liew | 2013 | 26 | 53 | Ertapenem,  imipenem, and meropenem (Current hospitalization) | Adjusted OR | 10.63 | 1.88 | 59.94 | <0.01 |
| Ustun | 2016 | 75 | 150 | *Carbapenems not specified* (time NR) | Adjusted RR | 4.92 | 1.6 | 15.09 | 0.005 |
| Cephalosporin |  |  |  |  |  |  |  |  |  |
| Aloush | 2006 | 82 | 82 | Broad spectrum cephalosporins (current hospitalization through study inclusion) | Adjusted OR | 9.9 | *2.178* | *44.997* | 0.003 |
| D'Agata (b) | 2006 | 151 | 151 | Cephalosporins (30 days prior to enrollment) | Adjusted OR | 3.5 | 1.7 | 7.1 | <0.001 |
| Willmann | 2014 | 31 | 93 | Ceftazidime use (time NR) | Adjusted OR | 1.9 | 0.22 | 16.43 | 0.56 |
| Aminoglycosides |  |  |  |  |  |  |  |  |  |
| Aloush | 2006 | 82 | 82 | Aminoglycosides use (current hospitalization through study inclusion) | Adjusted OR | 6.1 | 1.086 | 34.262 | 0.04 |
| D'Agata (b) | 2006 | 151 | 151 | Gentamicin (30 days prior to enrollment) | Adjusted OR | 2.3 | 1.04 | 5.1 | 0.04 |
| Cobos-Trigueros | 2015 | 31 | 819 | Amikacin >3 days | adj OR | 4.5 | 1.3 | 15.2 | 0.017 |
| Other Antibiotics |  |  |  |  |  |  |  |  |  |
| Defez | 2004 | 80 | 240 | Beta-lactams (7 days before PA isolation) | Adjusted OR | 2.5 | 1 | 6.3 | 0.04 |
| Montero (a) | 2010 | 345 | 690 NON PA | Anti-PA penicillin (time NR) | Adjusted OR | 1.09 | *0.527* | *2.253* | 0.816 |
| Montero (b) | 2010 | 345 | 532 | Anti-PA penicillin (time NR) | Adjusted OR | 2.79 | *1.075* | *7.242* | 0.035 |
| Dalfino | 2011 | NR | NR | Ampicillin/Sulbactam (< 2 previous weeks) | Adjusted OR | 3.2 | 1.3 | 5.2 | 0.042 |
| Dalfino | 2011 | NR | NR | Glycopeptides (< 2 previous weeks) | Adjusted OR | 5.3 | 2.8 | 7.2 | 0.008 |
| Dalfino | 2011 | NR | NR | Tigecycyline (< 2 previous weeks) | Adjusted OR | 4.2 | 2.3 | 6.8 | 0.041 |
| Nseir | 2011 | 82 | 429 | Piperacillin/tazobactam use (3 mo before ICU admission) | Adjusted OR | 1.2 | 1.1 | 1.3 | 0.04 |
| Gomez-Zorilla | 2014 | 23 | 89 | Piperacillin-tazobactam (3 mo before PA isolation) | Adjusted HR | 0.95 | 0.88 | 1.02 | 0.19 |
| Any prior antibiotic therapy |  |  |  |  |  |  |  |  |  |
| Cilloniz | 2016 | 22 | 46 | Prior antibiotic treatment (30 days before admission) | Adjusted OR | 3.32 | 1.07 | 10.31 | 0.038 |
| Tumbarello | 2011 | 40 | 212 | Previous antibiotic therapy (30 days before onset of infection) | Adjusted OR | 2.79 | 1.1 | 7.07 | 0.03 |
| Pena | 2012 | 13 | 95 | Days of prior antibiotic exposure (within 90 days before the onset of PA) | Adjusted OR | 1.1 | 1.02 | 1.2 | 0.01 |
| Number of antibiotics |  |  |  |  |  |  |  |  |  |
| Aloush | 2006 | 82 | 82 | Number of antibiotic classes | Adjusted OR | 1.8 | *1.151* | *2.815* | 0.01 |
| Lodise | 2007 | 122 | 229 | Number of prior antibiotic exposures, per unit increase | Adjusted OR | 1.3 | 1.2 | 1.5 | <0.001 |
| Ohmagari | 2005 | 18 | 36 | History of previous PA infection (time NR) | Adjusted OR | 13.7 | 1.79 | 111.1 | 0.12 |
| Willmann | 2014 | 31 | 93 | N different antibiotics >3 during time at risk | Adjusted OR | 2.22 | 0.36 | 13.65 | 0.39 |

HR = Hazard ratio; NR= not reported; OR = odds ratio

* Dalfino 2011, ICU stay was used for the control

Italicized CIs indicate values were calculated using estimate and P-value

**Table S4. Other** Treatment–related Multivariate Risk Factors of Acquistion of MDR and XDR P. aeruginosa

| Author | Year | N  Cases / Exposure | N comparator | Risk Factors /Predictors | Multivariate metric | Results | 95% LCI | 95% UCI | P-value |
| --- | --- | --- | --- | --- | --- | --- | --- | --- | --- |
| Surgery |  |  |  |  |  |  |  |  |  |
| Defez | 2004 | 80 | 75 | Surgery (hospitalized patients-type NR) | Adjusted OR | 0.5 | 0.2 | 0.98 | 0.04 |
| Nakamura | 2013 | 159 | 276 | Surgery (type NR) | Adjusted OR | 2.6 | 2.6 | 4.5 | <0.001 |
| Nseir | 2011 | 82 | 429 | Surgery (type NR) | adj OR | 1.9 | 1.1 | 3.6 | 0.024 |
| Cobos-Trigueros | 2015 | 31 | 819 | Emergency surgery prior to ICU admission | adj OR | 2.8 | 1.1 | 7.3 | 0.03 |
| Urinary catheter |  |  |  |  |  |  |  |  |  |
| Defez | 2004 | 80 | 240* | Urinary catheter (Infected with P. aeruginosa) | Adjusted OR | 4.1 | 1.7 | 9.6 | 0.0012 |
| Eagye (a) | 2009 | 58 | 125 | Use of Foley catheter | Adjusted OR | 2.53 | 1.18 | 5.45 | 0.018 |
| Joo | 2011 | 42 | 160 | Indwelling urinary catheter | Adjusted OR | 4.28 | 1.14 | 5.53 | 0.001 |
| Liew (XDR) | 2013 | 26 | 53 | Foley catheter insertion | Adjusted OR | 11.24 | 1.08 | 117.62 | 0.04 |
| Willmann (XDR) | 2014 | 31 | 93 | Urinary catheter | Adjusted OR | 21.04 | 3.67 | 120.57 | <0.0001 |
| Other devices |  |  |  |  |  |  |  |  |  |
| Cao | 2004 | 44 | 68 | Mechanical ventilation | Adjusted OR | 8.19 | 1.65 | 40.7 | 0.01 |
| Defez | 2004 | 80 | 240* | Nasogastric feeding (Infected with P. aeruginosa) | Adjusted OR | 5.3 | 1.4 | 20.5 | 0.016 |
| Cobos-Trigueros | 2015 | 31 | 819 | Nasogastric tube >3days | Adjusted OR | 3.3 | 1.3 | 8.5 | 0.014 |
| Aloush | 2006 | 82 | 82 | High invasive devices score | Adjusted OR | 13.9 | NR | NR | 0.02 |
| Johnson | 2009 | 113 | 390 | Previous transplantation | Adjusted OR | 2.38 | 1.51 | 3.76 | <0.001 |
| Bodro  (XDR) | 2015 | 31 strains (24 patients) | 318 | Prior transplantation | Adjusted OR | 5.2 | 1.6 | 16.8 | 0.006 |
| Joo | 2011 | 42 |  | Percutaneous catheterization | Adjusted OR | 2.51 | 1.14 | 5.53 | 0.023 |
| Nakamura | 2013 | 159 | 276 | Urinary specimens | Adjusted OR | 7.3 | 3.5 | 15.1 | <0.001 |
| Dalfino | 2011 | NR | NR | Total parenteral nutrition | Adjusted OR | 2.7 | 1.3 | 4.2 | 0.0001 |
| Cobos-Trigueros | 2015 | 31 | 819 | Endoscopy | Adjusted OR | 3.7 | 1.6 | 8.5 | 0.002 |

NR = not reported; OR = odds ratio

* random controls

**Table S5. Hospital**–related Multivariate Risk Factors of Acquistion of MDR and XDR P. aeruginosa

| Author | Year | N  Cases / Exposure | N comparator | Risk Factors /Predictors | Multivariate metric | Results | 95% LCI | 95% UCI | P-value |
| --- | --- | --- | --- | --- | --- | --- | --- | --- | --- |
| ICU stay |  |  |  |  |  |  |  |  |  |
| Aloush | 2006 | 82 | 82 | ICU stay | Adjusted OR | 10.1 | *1.111* | *91.791* | 0.04 |
| Eagye (b) | 2009 | 58 | 57 | More prior ICU days | Adjusted OR | 1.1 | 1.03 | 1.18 | 0.005 |
| Johnson | 2009 | 113 | 212 | ICU admission in previous year | Adjusted OR | 2.04 | 1.15 | 3.63 | NR |
| Johnson | 2009 | 55 | 94 | ICU admission in previous year | Adjusted OR | 3.82 | 1.22 | 12 | NR |
| Tuncer | 2012 | 37 | 83 | Neurology ICU | Adjusted OR | 3.57 | 1.38 | 9.18 | 0.008 |
| Dantas | 2014 | 57 | 63 | ICU | Adjusted OR | 3.28 | *1.206* | *8.923* | 0.02 |
| Micek | 2015 | 226 | 514 | ICU admission | Adjusted OR | 1.73 | 1.06 | 2.81 | 0.028 |
| Prior Hospitalization |  |  |  |  |  |  |  |  |  |
| Eagye (a) | 2009 | 58 | 125 | More prior admissions (prior 12 months) | Adjusted OR | 1.41 | 1.15 | 1.74 | 0.001 |
| Eagye (b) | 2009 | 58 | 57 | More prior admissions (prior 12 months) | Adjusted OR | 1.4 | 1.08 | 1.81 | 0.01 |
| Montero (a) | 2010 | 345 | 690 (non-PA) | Previous hospitalization >/= 3 | Adjusted OR | 3.52 | *1.66* | *7.54* | <0.001 |
| Montero (a) | 2010 | 345 | 690 (non-PA) | Previous hospitalization 1 | Adjusted OR | 1.3 | *0.85* | *1.99* | 0.226 |
| Montero (a) | 2010 | 345 | 690 (non-PA) | Previous hospitalization 2 | Adjusted OR | 2.15 | *1.36* | *3.39* | 0.001 |
| Montero (b) | 2010 | 345 | 532 (SPA) | Previous hospitalization >/= 3 | Adjusted OR | 2.87 | *1.53* | *5.35* | <0.001 |
| Montero (b) | 2010 | 345 | 532 (SPA) | Previous hospitalization 1 | Adjusted OR | 1.57 | *0.97* | *2.54* | 0.065 |
| Montero (b) | 2010 | 345 | 532 (SPA) | Previous hospitalization 2 | Adjusted OR | 2.86 | *1.53* | *5.35* | <0.001 |
| Time at risk |  |  |  |  |  |  |  |  |  |
| Defez | 2004 | 80 | 75 | Time at risk = 33 days | Adjusted OR | 1.9 | 0.8 | 4.5 | 0.12 |
| Eagye (b) | 2009 | 58 | 57 | Increased time at risk | Adjusted OR | 1.01 | 0.98 | 1.03 | 0.675 |
| Eagye (a) | 2009 | 58 | 125 | Increase in time at risk | Adjusted OR | 1.03 | 1.01 | 1.06 | 0.004 |
| Dalfino | 2011 | NR | NR | Time to risk (time elapsed from ICU admission to isolation of MDR PA); Length of ICU stay for controls | Adjusted OR | 3.8 | 1.7 | 5.2 | 0.009 |
| LOS prior to culture |  |  |  |  |  |  |  |  |  |
| Lodise | 2007 | 122 | 229 | LOS prior to culture >=33 days | Adjusted OR | 1.9 | 1.3 | 2.6 | <0.001 |
| Nakamura | 2013 | 159 | 276 | Length of hospital stay (time NR) | Adjusted OR | NR |  |  | <0.001 |
| Ustun | 2016 | 75 | 150 | Long hospitalization>10 days | adj RR | 4.68 | 2.09 | 10.49 | <0.001 |
| Hospital-acquired |  |  |  |  |  |  |  |  |  |
| Johnson | 2009 | 113 | 390 (non-MDR) | Hosptial-acquired BSI | Adjusted OR | 2.41 | 1.39 | 4.18 | <0.001 |
| Johnson | 2009 | 55 | 94 (non-MDR transplant) | Hosptial-acquired BSI | Adjusted OR | 2.95 | 1.21 | 7.16 | 0.002 |
| Pena | 2009 | 162 | 84 | Nosocomial acquisition | Adjusted OR | 2.7 | 1.1 | 6.3 | NR |
| Bodro  (XDR) | 2015 | 31 strains (24 patients) | 318 | Nosocomial acquisition | Adjusted OR | 7 | 1.5 | 30.7 | 0.01 |
| Others |  |  |  |  |  |  |  |  |  |
| Defez | 2004 | 80 | 240* | Transfer from another care unit (Infected with P. aeruginosa) | Adjusted OR | 3.9 | 1.4 | 11.4 | 0.011 |
| Defez | 2004 | 80 | 75 | Transfer from another care unit (Hospitalized patients) | Adjusted OR | 1.9 | 0.8 | 4.2 | 0.12 |
| Ohmagari | 2005 | 18 | 36 | History of previous PA infection (time NR) | Adjusted OR | 13.7 | 1.79 | 111.1 | 0.12 |
| Nseir | 2011 | 82 | 429 | Prior occupant with MDR PA | Adjusted OR | 2.3 | 1.2 | 4.3 | 0.012 |
| Pena | 2012 | 13 | 95 | Prior colonization with clinical samples other than blood isolates (time NR) | Adjusted OR | 8.4 | 0.76 | 93.2 | 0.058 |
| Gomez-Zorilla | 2014 | 23 | 89 | Prior non-MDR colonization (time NR) | Adjusted HR | 0.61 | 0.21 | 1.75 | 0.36 |
| Dantas (MDR) | 2014 | 57 | 63 | Respiratory tract source of BSI | Adjusted OR | 5.38 | *1.303* | *22.207* | 0.02 |
| Dantas  (XDR) | 2014 | 31 | 89 | Respiratory tract source of BSI | Adjusted OR | 3.54 | *1.000* | *12.532* | 0.05 |

* MDR P.aeruginosa vs non-nosocomial MDR P. aeruginosa

BSI = Blood stream infection; ICU = Intensive care unit; HR = hazard ratio; OR = odds ratio

Italicized CIs indicate values were calculated using estimate and P-value

**Table S6. Patient –related Multivariate Risk Factors of Acquistion of Carbapenem-resistant P. aeruginosa**

| **Author** | **Year** | **N**  **Cases / Exposure** | **N comparator** | **Risk Factors /Predictors** | **Multivariate metric** | **Results** | **95% LCI** | **95% UCI** | **P-value** |
| --- | --- | --- | --- | --- | --- | --- | --- | --- | --- |
| **CRPA vs. CSPA** |  |  |  |  |  |  |  |  |  |
| Djordjevic | 2013 | 167 | 94 | Male gender | Adjusted OR | 2.313 | 1.173 | 4.562 | 0.016 |
| DalBen | 2013 | 67* | 258 | Male gender | Adjusted OR | 2.24 | 1.24 | 4.05 | 0.008 |
| Pena | 2007 | 42 | 254 | Severity of acute illness according to SAPS | Adjusted OR | 1 | 1 | 1.1 | NR |
| DalBen | 2013 | 67* | 258 | APACHE II score | Adjusted OR | 1.11 | 1.06 | 1.16 | <0.001 |
| Lee | 2015 | 25 | 50 | Pittsburgh score | Adjusted OR | 1.361 | 0.974 | 1.903 | 0.071 |
| D'Agata (a) | 2006 | 41 | 41 | Nonambulatory status | Adjusted OR | 5.6 | 1.4 | 23 | 0.02 |
| Lee | 2015 | 25 | 50 | Creatinine (threshold NR) | Adjusted OR | 0.558 | 0.282 | 1.102 | 0.093 |
| Zavascki(b) | 2005 | 93 | 65 | Renal failure | Adjusted OR | 5 | 1.28 | 19.53 | 0.02 |
| Lee | 2015 | 25 | 50 | Platelet count (threshold NR) | Adjusted OR | 1 | 0.094 | 1.007 | 0.938 |
| **IRPA vs ISPA** |  |  |  |  |  |  |  |  |  |
| Furtado | 2010 | 58 | 237 | Male gender | Adjusted OR | 8.01 | 1.66 | 38.51 | 0.009 |
| Furtado | 2010 | 58 | 237 | APACHE II score | Adjusted OR | 1.11 | 1.01 | 1.22 | 0.003 |

* includes meropenem-resistant Acinetobacter

CI= confidence interval; CRPA = carbapenem-resistant P. aeruginosa; CSPA = carbapenem-sensitive P. aeruginosa; NR = not reported; OR = odds ratio

**Table S7. Antibiotic Treatment–related Multivariate Risk Factors of Acquistion of Carbapenem-resistant P. aeruginosa**

| Author | Year | N  Cases / Exposure | N comparator | Risk Factors /Predictors | Multivariate metric | Results | 95% LCI | 95% UCI | P-value |
| --- | --- | --- | --- | --- | --- | --- | --- | --- | --- |
| CRPA vs. CSPA |  |  |  |  |  |  |  |  |  |
| Any Carbapenems |  |  |  |  |  |  |  |  |  |
| Pena | 2007 | 42 | 254 | Any carbapenem use (time NR) | Adjusted OR | 7.8 | 1.7 | 35.3 |  |
| Tuon | 2012 | 29 | 48 | Carbapenem during current stay (including ertapenem) | Adjusted OR |  |  |  | 0.014 |
| Luyt | 2014 | 68 | 101 | Carbapenem use (Groups I&II within 15 days) | Adjusted OR | 3.74 | 1.88 | 7.45 | NR |
| Lee | 2015 | 25 | 50 | Carbapenem (Groups I&II within 3 months) | Adjusted OR | 360.72 | 8.101 | 16061.83 | 0.002 |
| Group II carbapenems |  |  |  |  |  |  |  |  |  |
| Djordjevic | 2013 | 167 | 94 | Prior use of imipenem (14 days prior hospitalization) | Adjusted OR | 0.174 | 0.046 | 0.659 | 0.01 |
| Djordjevic | 2013 | 167 | 94 | Prior use of meropenem (14 days prior hospitalization) | Adjusted OR | 0.668 | 0.269 | 1.659 | 0.384 |
| Luyt | 2014 | 68 | 101 | Meropenem use (time NR) | Adjusted OR | 3.38 | 1.68 | 6.8 | NR |
| Quinolones |  |  |  |  |  |  |  |  |  |
| Lin | 2016 | 82 | 82 | Prior receipt of fluoroquinolones (last 15 days) | Adjusted OR | 4.64 | 1.64 | 13.14 | 0.004 |
| Pena | 2007 | 42 | 254 | Prior use fluoroquinolones (time NR) | Adjusted OR | 11 | 1.7 | 67.9 |  |
| Kohlenberg | 2010 | 15 | 18 | Quinolone therapy (time NR) | Adjusted OR | 48.37 | 3.71 | 999 | NR |
| Djordjevic | 2013 | 167 | 94 | Prior use of ciprofloxacin (14 days before hospitalization) | Adjusted OR | 0.68 | 0.329 | 1.408 | 0.299 |
| Other antibiotics |  |  |  |  |  |  |  |  |  |
| Fortaleza (b) | 2006 | 55 | 110 | Amikacin use (time NR) | Adjusted OR | 3.69 | 1.32 | 10.35 | 0.01 |
| Djordjevic | 2013 | 167 | 94 | Prior use of amikacin (14 days prior hospitalization) | Adjusted OR | 0.736 | 0.38 | 1.424 | 0.362 |
| Djordjevic | 2013 | 167 | 94 | Prior use of piperacillin+tazobactam (14 days prior hospitalization) | Adjusted OR | 1.422 | 0.552 | 3.669 | 0.466 |
| Djordjevic | 2013 | 167 | 94 | Prior use of ceftazidime (14 days prior hospitalization) | Adjusted OR | 0.852 | 0.368 | 1.971 | 0.708 |
| Lee | 2015 | 25 | 50 | Antipseudomonal cephalosporins within 3 mo | Adjusted OR | 0.237 | 0.017 | 3.275 | 0.283 |
| Djordjevic | 2013 | 167 | 94 | Prior use of vancomycin (14 days prior hospitalization) | Adjusted OR | 0.964 | 0.357 | 2.606 | 0.942 |
| Lee | 2015 | 25 | 50 | Glycopeptides within 3 months | Adjusted OR | 2.182 | 0.339 | 14.024 | 0.411 |
| Djordjevic | 2013 | 167 | 94 | Antibiotics used in the last month | Adjusted OR | 0.848 | 0.408 | 1.76 | 0.657 |
| IRPA vs ISPA |  |  |  |  |  |  |  |  |  |
| Any carbapenem |  |  |  |  |  |  |  |  |  |
| Zavascki (a) | 2005 | 93 | 93* | carbapenem use irrespective of vancomycin (14 days prior PA isolation) | Adjusted OR | 5.82 | 2.41 | 4.07 | <0.001 |
| Zavascki (a) | 2005 | 93 | 93* | Carbapenem use without vancomycin (14 days prior PA isolation) | Adjusted OR | 3.57 | 1.38 | 9.19 | 0.008 |
|  |  |  |  |  |  |  |  |  |  |
| Zavascki (b) | 2005 | 93 | 65 (ISPA) | carbapenem use irrespective of vancomycin (14 days prior PA isolation) | Adjusted OR | 12.82 | 3.99 | 41.23 | <0.001 |
| Lautenbach | 2010 | 253 | 2289 | Carbapenem use (within prior 30 days) | Adjusted OR | 7.92 | 4.78 | 13.11 | <0.001 |
| Any carbapenem with vancomycin |  |  |  |  |  |  |  |  |  |
| Zavascki (a) | 2005 | 93 | 93* | Carbapenem use with vancomycin (14 days prior PA isolation) | Adjusted OR | 43.71 | 4.46 | 428.53 | <0.001 |
| Onguru | 2008 | 75 | 95 | Previous use of combination of vancomycin and imipenem (14 days prior PA isolation) | Adjusted OR | 10.909 | 1.332 | 89.351 | 0.0001 |
| Group II carbapenems |  |  |  |  |  |  |  |  |  |
| Harris | 2002 | 120 | 746 | Exposure to imipenem (14 days prior PA isolation) | Adjusted OR | 4.96 | 2.88 | 8.57 | <0.0001 |
| Fortaleza (a) | 2006 | 108 | 216 | Imipenem use (time NR) | Adjusted OR | 18.51 | 6.3 | 54043 | <0.001 |
| Onguru | 2008 | 75 | 95 | Previous use of imipenem tazobactam (14 days prior PA isolation) | Adjusted OR | 3.58 | 1.252 | 10.245 | 0.017 |
| Harris | 2011 | 118 | 3028 | Imipenem exposure (14 days prior PA isolation) | Adjusted OR | 6.06 | 3.97 | 9.24 | NR |
| Vancomycin |  |  |  |  |  |  |  |  |  |
| Harris | 2002 | 120 | 746 | exposure to vancomycin (14 days prior PA isolation) | Adjusted OR | 1.8 | 1.09 | 2.96 | 0.02 |
| Fortaleza (a) | 2006 | 108 | 216 | Vancomycin use (time NR) | Adjusted OR | 2.48 | 1.08 | 5.64 | 0.03 |
| Onguru | 2008 | 75 | 95 | Pervious use of vancomycin tazobactam (14 days prior PA isolation) | Adjusted OR | 2.882 | 1.13 | 7.349 | 0.027 |
| Piperacillin-tazobactam |  |  |  |  |  |  |  |  |  |
| Harris | 2002 | 120 | 746 | Exposure to Piperacillin-tazobactam (14 days prior PA isolation) | Adjusted OR | 2.39 | 1.42 | 4.03 | 0.0011 |
| Onguru | 2008 | 75 | 95 | Previous use of piperacillin-tazobactam tazobactam (14 days prior PA isolation) | Adjusted OR | 6.425 | 2.187 | 18.875 | 0.001 |
| Furtado | 2010 | 58 | 237 | Use of piperacillin-tazobactam tazobactam (3 weeks prior PA isolation) | Adjusted OR | 14.31 | 1.02 | 200.16 | 0.04 |
| Other antibiotics |  |  |  |  |  |  |  |  |  |
| Harris | 2002 | 120 | 746 | exposure to Aminoglycoside (14 days prior PA isolation) | Adjusted OR | 2.19 | 1.35 | 3.56 | 0.0015 |
| Fortaleza (a) | 2006 | 108 | 216 | Amikacin use (time NR) | Adjusted OR | 3.22 | 1.4 | 7.41 | 0.005 |
| Furtado | 2010 | 58 | 237 | Use of 3rd-generation cephalosporin (3 weeks prior PA isolation) | Adjusted OR | 7.45 | 1.8 | 30.86 | 0.006 |
| Furtado | 2010 | 63 | 182 | No. of antibiotics used | Adjusted OR | 1.38 | 0.97 | 1.97 | 0.07 |

* non-P.aeruginosa

CI= confidence interval; CRPA = carbapenem-resistant P. aeruginosa; CSPA = carbapenem-sensitive P. aeruginosa; NR = not reported; OR = odds ratio

**Table S8. Other Treatment–related Multivariate Risk Factors of Acquistion of Carbapenem-resistant P. aeruginosa**

| **Author** | **Year** | **N**  **Cases / Exposure** | **N comparator** | **Risk Factors /Predictors** | **Multivariate metric** | **Results** | **95% LCI** | **95% UCI** | **P-value** |
| --- | --- | --- | --- | --- | --- | --- | --- | --- | --- |
| **CRPA vs. CSPA** |  |  |  |  |  |  |  |  |  |
| Djordjevic | 2013 | 167 | 94 | Surgery (type NR) | Adjusted OR | 0.7 | 0.366 | 1.337 | 0.28 |
| Djordjevic | 2013 | 167 | 94 | Urinary catheter >7 days | Adjusted OR | 0.435 | 0.214 | 0.882 | 0.021 |
| Djordjevic | 2013 | 167 | 94 | Central venous catheter | Adjusted OR | 0.453 | 0.193 | 1.062 | 0.069 |
| Djordjevic | 2013 | 167 | 94 | Mechanical ventilation | Adjusted OR | 1.865 | 0.767 | 4.53 | 0.169 |
| Khayr | 2000 | 42 | 52 | Indwelling airway | Adjusted OR | 10.5 | NR | NR | 0.009 |
| Luyt | 2014 | 68 | 101 | Mechanical ventilation | Adjusted OR | 1.02 | 1.01 | 1.05 | NR |
| Kohlenberg | 2010 | 15 | 18 | Abdominal and/or thoracic drain | Adjusted OR | 64.33 | 5.32 | 999 | NR |
| DalBen | 2013 | 67* | 258 | Surgery prior to admission (type NR) | Adjusted OR | 0.29 | 0.13 | 0.65 | 0.003 |
| Lee | 2015 | 25 | 50 | Hospital stay (before bacteremia onset) | Adjusted OR | 0.97 | 0.93 | 1.01 | 0.140 |
| **IRPA vs ISPA** |  |  |  |  |  |  |  |  |  |
| Zavascki(a) | 2005 | 93 | 93** | Mechanical ventilation | Adjusted OR | 3.22 | 1.52 | 6.83 | 0.002 |
| Pereira | 2008 | 30 | 29 | urinary catheter >15 days (collinear variable) | Adjusted OR | 5.95 | 1.59 | 22.33 | <0.01 |
| Onguru | 2008 | 75 | 95 | Arterial line | Adjusted OR | 2.508 | 1.062 | 5.92 | 0.036 |
| Furtado | 2010 | 58 | 237 | Hemodialysis | Adjusted OR | 6.85 | 1.33 | 35.2 | 0.02 |
| Fortaleza (a) | 2006 | 108 | 216 | Hemodialysis | Adjusted OR | 7.79 | 1.59 | 38.16 | 0.01 |
| Furtado | 2010 | 58 | 237 | Use of corticosteriod | Adjusted OR | 13.18 | 3.8 | 45.64 | <0.001 |

* includes meropenem-resistant Acinetobacter

** non-P.aeruginosa

CI= confidence interval; CRPA = carbapenem-resistant P. aeruginosa; CSPA = carbapenem-sensitive P. aeruginosa; IRPA = imipenem-resistant P. aeruginosa; ISPA = imipenem-sensitive P. aeruginosa; NR = not reported; OR = odds ratio

**Table S9. Hospital–related Multivariate Risk Factors of Acquistion of Carbapenem-resistant P. aeruginosa**

| **Author** | **Year** | **N**  **Cases / Exposure** | **N comparator** | **Risk Factors /Predictors** | **Multivariate metric** | **Results** | **95% LCI** | **95% UCI** | **P-value** |
| --- | --- | --- | --- | --- | --- | --- | --- | --- | --- |
| **CRPA vs. CSPA** |  |  |  |  |  |  |  |  |  |
| DalBen | 2013 | 67 | 258 | Colonization pressure in the week before the outcomes | Adjusted OR | 1.02 | 1.01 | 1.04 | 0.008 |
| DalBen | 2013 | 67 | 258 | Surgery prior to admission | Adjusted OR | 0.29 | 0.13 | 0.65 | 0.003 |
| Djordjevic | 2013 | 167 | 94 | ICU stay (time NR) | Adjusted OR | 0.7 | 0.366 | 1.337 | 0.28 |
| Djordjevic | 2013 | 167 | 94 | ICU stay (time NR) | Adjusted OR | 0.455 | 0.209 | 0.99 | 0.047 |
| Lee | 2015 | 25 | 50 | Length of hospital stay | Adjusted OR | 0.97 | 0.932 | 1.01 | 0.14 |
| **IRPA vs ISPA** |  |  |  |  |  |  |  |  |  |
| Harris | 2002 | 120 | 746 | ICU stay (before PA isolation) | Adjusted OR | 3.26 | 1.82 | 5.87 | <0.0001 |
| Furtado* | 2010 | 63 | 182 | Previous ICU stay (before PA isolation) | Adjusted OR | 3.54 | 1.29 | 9.73 | 0.03 |
| Onguru | 2008 | 75 | 95 | Hospital stay until infection (before onset of bacteremia) | Adjusted OR | 1.027 | 1.002 | 1.054 | 0.034 |
| Pereira | 2008 | 30 | 29 | Hospital stay >15 days | Adjusted OR | 6.7 | 1.86 | 24.14 | <0.01 |
| Harris | 2002 | 120 | 746 | Time at risk (admission to PA isolation) | Adjusted OR | 1.02 | 1.01 | 1.04 | 0.0006 |
| Harris | 2011 | 118 | 3028 | Time at risk (>4.6 days) | Adjusted OR | 2 | 1.27 | 3.16 | NR |
| Zavascki(a) | 2005 | 93 | 93 | At least one admission in last year | Adjusted OR | 2.59 | 1.2 | 5.56 | 0.015 |
| Fortaleza (a) | 2006 | 108 | 216 | Transfer from another hospital | Adjusted OR | 4.21 | 1.4 | 12.66 | 0.01 |
| Lautenbach | 2010 | 253 | 2289 | Transfer from another facility | Adjusted OR | 1.77 | 1.33 | 2.37 | <0.001 |
| Furtado | 2010 | 58 | 237 | Length of hospital stay | Adjusted OR | 1.19 | 1.12 | 1.26 | <0.001 |
| Lautenbach | 2010 | 253 | 2289 | Length of hospital stay prior to culture sampling | Adjusted OR | 1.01 | 1 | 1.01 | 0.03 |
| Harris | 2011 | 118 | 3028 | Colonization pressure (>3.2%) | Adjusted OR | 9.58 | 5.09 | 18.05 | NR |

CI= confidence interval; CRPA = carbapenem-resistant P. aeruginosa; CSPA = carbapenem-sensitive P. aeruginosa; IRPA = imipenem-resistant P. aeruginosa; ISPA = imipenem-sensitive P. aeruginosa; NR = not reported; OR = odds ratio

**Table S10. Multivariate Risk Factors of Acquistion of Resistant P. aeruginosa**

| **Author** | **Year** | **N**  **Cases / Exposure** | **N comparator** | **Risk Factors /Predictors** | **Multivariate metric** | **Results** | **95% LCI** | **95% UCI** | **P-value** |
| --- | --- | --- | --- | --- | --- | --- | --- | --- | --- |
| **Piperacillin-resistant** |  |  |  |  |  |  |  |  |  |
| Trouillet | 2002 | 34 | 101 | Underlying medical condition that is rapidly or ultimately fatal | Adjusted OR | 5.6 | 2 | 16.2 | 0.001 |
| Trouillet | 2002 | 34 | 101 | Previous exposure to fluoroquinolone (15 days prior to PA isolation) | Adjusted OR | 4.6 | 1.7 | 12.7 | 0.003 |
| Trouillet | 2002 | 34 | 101 | APACHE II score, per point accorded | Adjusted OR | 0.8 | 0.7 | 0.9 | <0.001 |
| **Piperacillin+ tazobactam resistant** |  |  |  |  |  |  |  |  |  |
| Harris | 2002 | 179 | 1136 | Piperacillin-tazobactam use (14 days prior to PA isolation) | Adjusted OR | 6.82 | 4.56 | 10.21 |  |
| Harris | 2002 | 179 | 1136 | Imipenem use (14 days prior to PA isolation) | Adjusted OR | 2.42 | 1.19 | 4.94 |  |
| Harris | 2002 | 179 | 1136 | Aminoglycoside use (14 days prior to PA isolation) | Adjusted OR | 2.18 | 1.44 | 3.28 |  |
| Harris | 2002 | 179 | 1136 | Vancomycin use (14 days prior to PA isolation) | Adjusted OR | 1.87 | 1.21 | 2.89 |  |
| Harris | 2002 | 179 | 1136 | Broad-spectrum cephalosporin use (14 days prior to PA isolation) | Adjusted OR | 2.38 | 1.45 | 3.88 |  |
| Gasink | 2006 | 320 | 527 | Prior fluoroquinolone use (30 days prior to PA isolation) | Adjusted OR | 3.43 | 2.37 | 4.96 | <0.001 |
| Hsu | 2005 | 91 | 86 | Prior fluoroquinolone exposure (30 days prior to PA isolation) | Adjusted OR | 12.6 | 4.9 | 32.2 | <0.005 |
| Harris | 2002 | 179 | 1136 | Admissions in prior year | Adjusted OR | 1.36 | 1.2 | 1.54 |  |
| Harris | 2002 | 179 | 1136 | ICU stay | Adjusted OR | 2.59 | 1.65 | 4.06 |  |
| Harris | 2002 | 179 | 1136 | Transfer from another facility | Adjusted OR | 2.07 | 1.31 | 3.27 |  |
| Harris | 2002 | 179 | 1136 | Time at risk | Adjusted OR | 1.02 | 1.01 | 1.03 |  |
| Gasink | 2006 | 320 | 527 | Hosptial days from admission to culture | Adjusted OR | 1 | 1 | 1.01 | 0.34 |
| Hsu | 2005 | 91 | 86 | Nosocomial residence | Adjusted OR | 8.6 | 3.5 | 20.7 | <0.005 |
| Hsu | 2005 | 91 | 86 | Comorbid diabetes mellitus | Adjusted OR | 6.4 | 2.1 | 19.3 | 0.001 |
| **Cefepime resistant** |  |  |  |  |  |  |  |  |  |
| Akhabue | 2011 | 213 | 2316 | Prior use of extended spectrum cephalosporin (30 days before PA isolation) | Adjusted OR | 2.18 | 1.57 | 3.04 | <0.001 |
| Akhabue | 2011 | 213 | 2316 | Prior use of extended spectrum penicillin (30 days before PA isolation) | Adjusted OR | 1.91 | 1.22 | 2.99 | 0.005 |
| Akhabue | 2011 | 213 | 2316 | Prior use of quinolone (30 days before PA isolation) | Adjusted OR | 1.96 | 1.38 | 2.78 | <0.001 |
| Akhabue | 2011 | 213 | 2316 | Prior use of carbapenem (30 days before PA isolation) | Adjusted OR | 1.7 | 0.9 | 3.21 | 0.1 |
| Akhabue | 2011 | 213 | 2316 | Transfer from outside facility | Adjusted OR | 1.49 | 1.09 | 2.04 | 0.01 |
| Akhabue | 2011 | 213 | 2316 | Length of hospital stay before culture | Adjusted OR | 1 | 0.99 | 1.01 | 0.81 |
| **Ciprofloxacillin-resistant** |  |  |  |  |  |  |  |  |  |
| Khayr | 2000 | 42 | 52 | Prior receipt of ciprofloxacin (time NR) | Adjusted OR | 16.8 | NR | NR | 0.0001 |
| D'Agata (a) | 2006 | 41 | 41  (non-P.aeruginosa) | Quinolones (30 days before study enrollment) | Adjusted OR | 5 | 1.2 | 21 | 0.03 |

CI= confidence interval; CRPA = carbapenem-resistant P. aeruginosa; CSPA = carbapenem-sensitive P. aeruginosa; IRPA = imipenem-resistant P. aeruginosa; ISPA = imipenem-sensitive P. aeruginosa; NR = not reported; OR = odds ratio

Figure S1. Meta-analysis of Risk Factors for Carbapenem versus Susceptible *P. aeruginosa* Acquisition


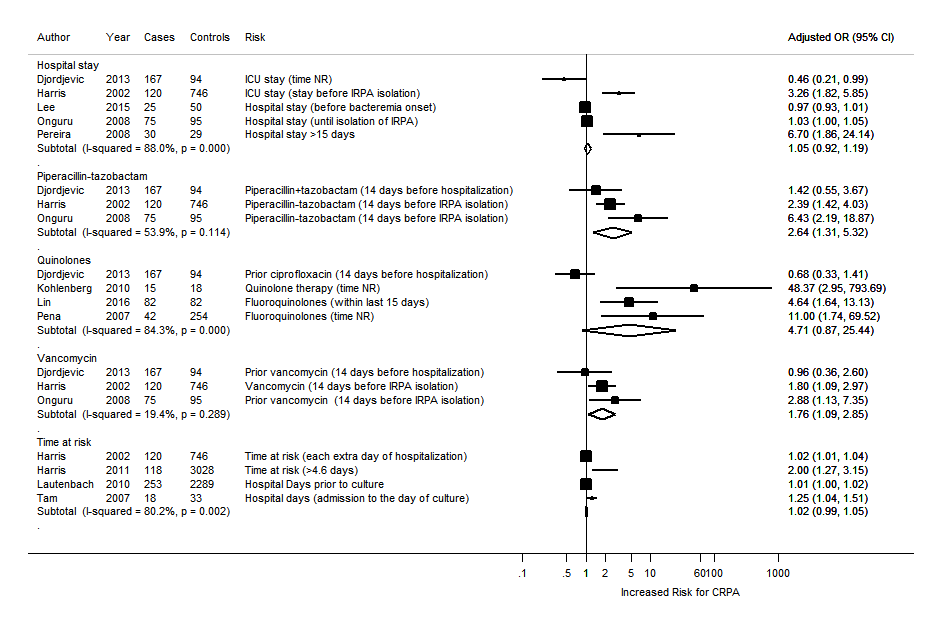


CI = confidence interval; CRPA = carbapenem-resistant *P.aeruginosa*; IRPA = imipenem-resistant *P.aeruginosa*; ICU = intensive care unit; NR = not reported; OR = odds ratio

Figure S2. Meta-analysis of Prior Use of Carbapenem as a Risk Factor for Carbapenem versus Susceptible *P. aeruginosa* Acquisition

CI = Confidence Interval; CRPA = carbapenem-resistant *P. aeruginosa*; IRPA = imipenem-resistant *P. aeruginosa*; NR = not reported; OR = odds ratio

Figure S3. Meta-analysis of Prior Use of Fluoroquinolones as a Risk Factor for Quinolone-resistant versus Susceptible *P. aeruginosa* Acquisition

CI = confidence interval; NR = not reported; OR = odds ratio
